# Whole genome genotyping reveals discrete genetic diversity in north‐east Atlantic maerl beds

**DOI:** 10.1111/eva.13219

**Published:** 2021-03-30

**Authors:** Tom L. Jenkins, Marie‐Laure Guillemin, Cornelia Simon‐Nutbrown, Heidi L. Burdett, Jamie R. Stevens, Viviana Peña

**Affiliations:** ^1^ Department of Biosciences, College of Life and Environmental Sciences University of Exeter Exeter UK; ^2^ Instituto de Ciencias Ambientales y Evolutivas, Facultad de Ciencias Universidad Austral de Chile Valdivia Chile; ^3^ IRL EBEA 3614, Evolutionary Biology and Ecology of Algae, CNRS, UC, UACH, Station Biologique de Roscoff Sorbonne Université Roscoff France; ^4^ Lyell Centre for Earth and Marine Science and Technology Edinburgh UK; ^5^ School of Energy, Geoscience, Infrastructure and Society Heriot‐Watt University Edinburgh UK; ^6^ Royal Botanic Garden Edinburgh Edinburgh UK; ^7^ BioCost Research Group, Facultad de Ciencias and Centro de Investigaciones Científicas Avanzadas (CICA) Universidade da Coruña, A Coruña Spain

**Keywords:** conservation management, coralline algae, maerl, mitogenome, plastome, population genetic structure, rhodolith, single nucleotide polymorphism

## Abstract

Maerl beds are vital habitats for a diverse array of marine species across trophic levels, but they are increasingly threatened by human activities and climate change. Furthermore, little is known about the genetic diversity of maerl‐forming species and the population structure of maerl beds, both of which are important for understanding the ability of these species to adapt to changing environments and for informing marine reserve planning. In this study, we used a whole genome genotyping approach to explore the population genomics of *Phymatolithon calcareum*, a maerl‐forming red algal species, whose geographical distribution spans the north‐east Atlantic, from Norway to Portugal. Our results, using 14,150 genome‐wide SNPs (single nucleotide polymorphisms), showed that *P. calcareum* maerl beds across the north‐east Atlantic are generally structured geographically, a pattern likely explained by low dispersal potential and limited connectivity between regions. Additionally, we found that *P. calcareum* from the Fal Estuary, south‐west England, is genetically distinct from all other *P. calcareum* sampled, even from The Manacles, a site located only 13 km away. Further analysis revealed that this finding is not the result of introgression from two closely related species, *Phymatolithon purpureum* or *Lithothamnion corallioides*. Instead, this unique diversity may have been shaped over time by geographical isolation of the Fal Estuary maerl bed and a lack of gene flow with other *P. calcareum* populations. The genomic data presented in this study suggest that *P. calcareum* genetic diversity has accumulated over large temporal and spatial scales, the preservation of which will be important for maximizing the resilience of this species to changes in climate and the environment. Moreover, our findings underline the importance of managing the conservation of maerl beds across western Europe as distinct units, at a site‐by‐site level.

## 1 INTRODUCTION

Free‐living unattached coralline red algae can aggregate on the seafloor to form extensive three‐dimensional habitats called maerl or rhodolith beds, found throughout the world's coastal oceans (Hernandez‐Kantun et al., 2017; Van Der Heijden & Kamenos, 2015). Across western Europe, maerl beds provide vital shelter in sublittoral zones for a myriad of animals and macroalgae (Barbera et al., 2003; Peña et al., 2014) and act as nursery areas for many species of fish and marine invertebrates (Kamenos et al., 2004; Kamenos et al., 2004; Pinho Costa et al., 2020). In addition, the role of coralline red algae as important carbon stores has been established, the production rates of which rival those of mangroves, salt marshes and seagrass beds (Mao et al., 2020; Van Der Heijden & Kamenos, 2015). However, maerl‐forming species are threatened by physical disturbances to the seabed and changes to the environment that impair photosynthesis and growth. For instance, maerl is highly sensitive to abrasion and sedimentation caused by resource extraction and coastal dredging (Bernard et al., 2019; Hall‐Spencer et al., 2003; Hall‐Spencer & Moore, 2000), and to local changes in seawater chemistry and temperature caused by eutrophication (Barbera et al., 2003), aquaculture (Hall‐Spencer et al., 2006), ocean acidification (Brodie et al., 2014; Noisette et al., 2013; Ragazzola et al., 2016) and climate change‐induced warming (Cornwall et al., 2019; Nelson, 2009; Qui‐Minet et al., 2019). Maerl is also extremely slow‐growing (<2 mm/year in Atlantic beds, Adey & McKibbin, 1970; Blake & Maggs, 2003) and has poor recovery potential (Hall‐Spencer & Moore, 2000). Therefore, if maerl beds are damaged and/or regress, this would inevitably reduce benthic biodiversity due to the loss of habitat and resources. Consequently, this has led to a number of international and national commitments in western Europe to protect maerl bed ecosystems across the north‐east Atlantic; for example, all European maerl beds (regardless of species) are listed as ‘Vulnerable’ on the European Red List of Habitats and are specifically acknowledged in the OSPAR list of threatened and/or declining habitats (OSPAR Commission, 2010).

In conservation biology, identifying distinct genetic diversity among populations and delineating biologically accurate management units are key objectives for marine and terrestrial management (Funk et al., 2012; Jenkins & Stevens, 2018; Palsbøll et al., 2007). However, despite the importance of maerl as ecosystem engineers (Sheehan et al., 2015), genetic diversity and population structure have only been investigated in one maerl‐forming species to‐date, *Phymatolithon calcareum* (Pardo et al., 2019). Using eight microsatellite loci, the study of Pardo *et al*. found clonality to be variable among western European maerl beds, even within the same region, and that genetic differentiation was generally high between most maerl beds studied; this supports the expectation that gametes, spores and clones of *P*. *calcareum* have low dispersal potential. The authors also reported that *P*. *calcareum* individuals were exclusively diploid sporophytes, although this could not be verified for samples from the Fal Estuary located in south‐west England. These latter samples were hypothesized to be possible triploids because of abnormal scoring patterns at three microsatellite loci, which the authors suggested could be linked to introgression, a phenomenon caused by hybridization between two species, that has recently been detected among macroalgal species within the same genus in both red algae (Savoie & Saunders, 2015) and brown algae (Montecinos et al., 2017) and has been found to occur in the wild between brown algal species from different families (Murúa et al., 2020).

Advances in technology and reduction in costs now permit population‐based studies of whole genome genotyping when dealing with species with relatively small genomes (<100 Mbp). In comparison to studies based on microsatellite markers, in which typically only tens of markers are isolated, whole genome genotyping can isolate thousands to tens of thousands of genetic markers across the genome of an organism, thereby providing unprecedented resolution into patterns of genetic diversity and population structure. In the present study, therefore, our first aim was to better understand population genetic structure in *P. calcareum* sampled from maerl beds across western Europe using genome‐wide single nucleotide polymorphisms (SNPs) isolated by whole genome genotyping. Our second aim was to investigate the hypothesis that introgression has occurred from a closely related species into the Fal Estuary using a subset of individuals and SNP loci. Specifically, we tested for evidence of introgression into *P. calcareum* from another maerl species belonging to the same genus, *P. purpureum*, and from *Lithothamnion corallioides*, a maerl species that, like *P. calcareum*, also forms extensive maerl beds in the Fal Estuary (Allen et al., 2014).

## 2 MATERIALS AND METHODS

### 2.1 Sampling and DNA extraction

Maerl samples were collected via scuba diving or snorkelling at 2–20 m depth from 12 sites across a 23° latitudinal range along the western European coast, from Norway to Portugal (Table 1). Maerl samples used in several previous studies (Carro et al., 2014; Pardo et al., 2014, 2019) were also used in this study. Additional maerl samples were collected for this study from (i) The Manacles in south‐west England, collected at 5–10 m depth via scuba diving in 2015 following guidelines from the Marine Management Organisation, and (ii) Loch Sween, western Scotland, collected at 2–3 m depth via snorkelling in 2019 (Table 1). To avoid the inclusion of potential clones in the present study, one individual was selected from one multilocus genotype (MLG) reported in the microsatellite‐based study (Pardo et al., 2019). Selecting one individual per microsatellite MLG was possible for all sites except the Fal Estuary, in which 10 samples were taken from a single MLG. This is because only two microsatellite MLGs were found at the Fal Estuary and only samples from the dominant MLG were available. In addition, five individuals of *Lithothamnion corallioides* identified in previous studies (Carro et al., 2014; Pardo et al., 2014) were also included in the present study to test whether this species has introgressed with *P. calcareum*. Two of these individuals were sampled from the Fal Estuary, while the remaining three individuals originated from France (Table 1). Sampling, DNA extraction and species verification protocols for these samples are outlined in the original papers (Table 1). For the samples collected from The Manacles, individuals were immediately placed in 95–100% ethanol and kept at 4°C for long‐term storage. Genomic DNA was extracted from The Manacles samples using the QIAGEN Blood and Tissue Kit following the manufacturer's protocol, including treatment with RNase A. Before digestion with proteinase K, each individual was cleaned of epiphytes using a scalpel and then carefully cut into small pieces. After an overnight digestion, the calcium carbonate skeleton was left in the tube, while the supernatant was pipetted to a new tube to proceed with the extraction protocol. The quality of DNA was assessed by using NanoDrop One purity ratios and by checking the molecular weight of each aliquot using gel electrophoresis on a 1% agarose gel; the quantity of DNA was assessed by fluorometry using the Qubit 1X dsDNA High Sensitivity Assay Kit. Loch Sween samples were kept in seawater overnight after collection and then visually cleaned of epiphytes and lysed using a QIAGEN Tissue Lyser II, and genomic DNA was extracted using the QIAGEN plant mini‐prep kit following manufacturer's protocols, including treatment with RNase A. Upon collection, these samples were thought to be *P. calcareum*; however, molecular analysis of organelle sequences during this study identified the DNA extracts as *P. purpureum*. The *cox1* mitochondrial and *psbA* plastid genes were extracted from the mitogenomes and plastomes, respectively, and BLASTn searches confirmed 100% matches to *P. purpureum* sequences on GenBank. As a result, the Loch Sween samples were only used in the introgression analysis.

**TABLE 1:** Sampling information

Site	Code	*N*	Year sampled	Lat	Lon	Depth (m)	Reference
*Phymatolithon calcareum*							
Norway, Hordaland	Nor	1	2013	60.541	4.846	7	Pardo et al. (2014)
Northern Ireland, Zara Shoal	Zar	12	2011	54.379	−5.564	10	Pardo et al. (2019)
Wales, Milford Haven	Mil	1	2011	51.704	−5.076	5–6	Pardo et al. (2019)
England, Fal Estuary	Fal	10	2011	50.164	−5.028	4–6	Pardo et al. (2019)
England, The Manacles	Man	6	2015	50.050	−5.050	5–10	This study
France, Morlaix	Mor	12	2011	48.711	−3.951	10–11	Pardo et al. (2019)
France, Trévignon	Tre	9	2011	47.795	−3.887	15	Pardo et al. (2019)
France, La Rochelle	Roc	2	2013	46.233	−1.381	12	Pardo et al. (2014)
Spain, Bornalle	Bor	12	2011	42.789	−9.020	11	Pardo et al. (2019)
Spain, Illa de Ons	Ons	11	2011	42.395	−8.915	13	Pardo et al. (2019)
Portugal, Armaçao de Pêra	Arm	2	2011	37.027	−8.317	20	Pardo et al. (2019)
*Phymatolithon purpureum*							
Scotland, Loch Sween	Sco / Ppur	12	2019	55.983	−5.632	2–3	This study
*Lithothamnion corallioides*							
England, Fal Estuary	Lcor05, Lcor06	2	2011	50.164	−5.028	4–6	Carro et al. (2014)
France, Morlaix	Lcor08	1	2011	48.711	−3.951	10–11	Carro et al. (2014)
France, Trévignon	Lcor09	1	2011	47.795	−3.887	15	Carro et al. (2014)
France, La Rochelle	Lcor11	1	2011	46.233	−1.381	12	Pardo et al. (2014)

Abbreviations: Lat, latitude (decimal degrees); Lon, longitude (decimal degrees); *N*, number of individuals sampled.

### 2.2 Whole genome sequencing

Red macroalgae genomes published to‐date vary in size from 43 Mbp (*Pyropia yezoensis*; Nakamura et al., 2013) to 105 Mbp (*Chondrus crispus*; Collén et al., 2013). As the genome size of *P. calcareum* is likely to fall within this range, a whole genome sequencing approach was employed for this study. The advantage of this approach over a reduced representation sequencing method, such as restriction site‐associated DNA sequencing (RADseq), is that all SNPs in the population sample can be identified and the cost is comparable to RADseq when working with small genomes. Genomic DNA was prepared in a skirted 96‐well plate and sent on dry ice to SNPsaurus (Oregen) for whole genome genotyping. Genomic DNA was first fragmented with Nextera reagent (Illumina, Inc), which also ligates short adapter sequences to the ends of the fragments; the Nextera reaction was scaled for fragmenting 5 ng of genomic DNA. The libraries were sequenced on a paired‐end 2x150 bp NovaSeq 6000 S4 lane.

### 2.3 *Phymatolithon calcareum* bioinformatics

DNA sequence reads were trimmed using Fastp 0.20.1 (Chen et al., 2018), and a *P*.* calcareum* reference was assembled *de novo* from one individual (Mor02) with default parameters using ABySS 2.0 (Jackman et al., 2017). A BLASTn search was conducted on the assembly and any contigs with hits to bacteria were removed. In addition, the Purge Haplotigs pipeline (Roach et al., 2018) was executed on the reference to flag potential haplotigs and identify and remove contigs with exceptionally low or high coverage that may represent assembly artefacts or organelle sequences. A *de novo* reference of adequate quality was not possible for *P. purpureum* or *L. corallioides* due to insufficient coverage of sequencing data for these samples. Trimmed reads from each sample were aligned to the *P. calcareum* reference using bwa‐mem2 (Vasimuddin et al., 2019). The alignment files were filtered using SAMtools 1.9 (Li et al., 2009) such that only properly paired mapped reads (‐f 0x2) that had a mapping quality score >30 were retained. Variants were called using BCFtools 1.9 (Li, 2011) and filtered using VCFtools 0.1.16 (Danecek et al., 2011).

Two data sets were created to target high‐quality discriminatory SNPs for (i) assessing population genetic structure in *P. calcareum* and (ii) investigating introgression. To generate a *P. calcareum* data set, first non‐*P. calcareum* samples and *P. calcareum* individuals which failed sequencing were removed. Second, SNPs were filtered such that remaining SNPs (i) were biallelic, (ii) had a minimum quality score of 30, (iii) had a minimum depth of 7, (iv) had a maximum depth of 100, (v) had no more than 5% missing data, (vi) had a minor allele count greater or equal to 5 and (vii) did not depart from Hardy–Weinberg equilibrium (alpha = 0.05). Third, where pairwise comparisons of SNP loci had a *r* ^2^ value of >0.50, only one locus was retained to mitigate the effects of linkage disequilibrium. Fourth, in the R programming environment (R Core Team, 2020), individuals that exceeded 20% missing data were removed and only polymorphic loci were retained for downstream analyses. Lastly, differentiation‐based outlier selection tests were conducted using the R package OutFLANK 0.2 (Whitlock & Lotterhos, 2015) to check for the presence of outlier loci (loci potentially under the influence of selection). OutFLANK calculates a likelihood on a trimmed distribution of *F*_ST_ values to infer the distribution of *F*_ST_ for neutral markers; it was executed using default parameters and an alpha of 0.05.

### 2.4 *Phymatolithon calcareum* data analysis

Although the inclusion of clones was mitigated by the experimental design (described in the sampling section above), the presence of clones was assessed *de novo*. Prevosti genetic distances were computed, and a heatmap and a histogram were visualized to assess the frequency of low genetic distances. The number of private alleles, allelic richness, observed heterozygosity and the inbreeding coefficient (*F*_IS_) were then calculated using the R packages poppr 2.8.6 (Kamvar et al., 2014) and hierfstat 0.5–7 (Goudet & Jombart, 2015). Genetic differentiation among sites was assessed by calculating pairwise Weir and Cockerham's *F*_ST_ (Weir & Cockerham, 1984); this metric was chosen because it is unbiased with respect to sample size when using thousands of biallelic SNP markers (Willing et al., 2012). Three methods were implemented to explore population structure and ancestral admixture. First, a principal component analysis (PCA) was performed using the *dudi*.*pca* function from adegenet 2.1.3 (Jombart & Ahmed, 2011). Second, admixture was estimated using the *snmf* function from LEA 3.2.0 (Frichot & François, 2015). This function uses sparse non‐negative matrix factorization (SNMF) to estimate individual ancestry coefficients from a genotype matrix. This analysis was chosen over other similar programs that estimate admixture because it is relatively insensitive to deviations from Hardy–Weinberg equilibrium and unequal sample sizes (Frichot & François, 2015), though sample sizes of two or less were excluded from this analysis to ensure accuracy. This analysis was run with 10 iterations for each *K*, and the cross‐entropy criterion was used to evaluate the optimal number of *K* ancestral populations to assume in the model. Third, a bifurcating maximum likelihood tree was built using TreeMix 1.13 (Pickrell & Pritchard, 2012). TreeMix uses population allele frequency data and estimates of genetic drift among sites using Gaussian approximation to build a tree, the branches of which represent the genetic relationships between sites. Migration events are then added in stepwise iterations for pairs of populations which are identified as poor fits to the tree model to maximize the likelihood until the explained variation of the model plateaus. TreeMix was run using the Fal Estuary as the root and up to seven migration events modelled.

### 2.5 Organelle genome extraction and analysis

As a comparison to the results gained from nuclear‐derived SNPs, mitochondrial and chloroplast genomes were assembled from the raw sequencing data. First, target organelle reads (either mitochondrial or plastid) were extracted using GetOrganelle 1.6.4 (Jin et al., 2020) using *P. calcareum* and *Lithothamnion sp*. sequences downloaded from GenBank as initial seeds (mitochondrion: KF808323 and MH281621; plastid: KC819266 and MH281627). Second, the target reads were assembled using Unicycler 0.4.9b (Wick et al., 2017), a program which expects the output assembly to be circular. All putative organelle contigs (circular and linear) were subjected to a BLASTn search to check that they matched the *Lithothamnion sp*. mitogenome or plastome reference on GenBank (MH281621 or MH281627, respectively). For each sample, the organelle genome sequence was used as input to GeSeq (Tillich et al., 2017), which predicts and annotates organelle genes captured in the assembly. For annotating mitogenomes, The Mold, Protozoan, and Coelenterate Mitochondrial Code and the Mycoplasma/Spiroplasma Code was used, while The Bacterial, Archaeal and Plant Plastid Code was used to annotate plastomes. For each sample, the coding sequences (CDS) predicted from GeSeq were extracted and concatenated using Geneious 6.1. In addition, to further confirm species identification, the mitochondrial *cox1* and plastid *psbA* CDS were compared to the NCBI GenBank database using a BLASTn search. Hits were filtered by percentage identity (95–100%) because only partial sequences of these CDS are present on Genbank (whereas the complete CDS are captured in the organelle genomes extracted here). The *cox1* and *psbA* CDS were chosen because they are the most commonly used markers in DNA barcoding and phylogenetic studies of maerl‐forming species (Melbourne et al., 2017; Pardo et al., 2014), and as a result, there is good representation of these sequences on GenBank. The concatenated CDS were then aligned using MAFFT 7.305 using default parameters. Lastly, Tamura and Nei (1993) pairwise genetic distances were computed and hierarchical clustering (UPGMA) was performed on the distance matrix. This analysis was performed using (i) an alignment containing only *P. calcareum* individuals and (ii) an alignment containing all individuals from each species.

### 2.6 Introgression analysis

To investigate potential introgression from *P. purpureum* or *L. corallioides* to *P. calcareum*, SNP filtering parameters were adjusted to optimize the number of high‐quality discriminatory SNPs retained. First, a subset of individuals was extracted, which included *L. corallioides* (Lcor), *P. purpureum* (Sco), all individuals of *P. calcareum* from the Fal Estuary (Fal) and The Manacles (Man), and one or two individuals of *P. calcareum* from each of Milford Haven (Mil), Morlaix (Mor), Norway (Nor), La Rochelle (Roc), Trévignon (Tre) and Zara Shoal (Zar). Second, SNPs were filtered such that remaining SNPs (i) were biallelic and polymorphic, (ii) had a minimum quality score of 30, (iii) had a minimum depth of 3 and (iv) had no more than 10% missing data. A PCA was then performed using the same method as described previously. Lastly, *snapclust* (Beugin et al., 2018) was run with the hybrid model activated (hybrids = TRUE) to test for a signature of introgression. This program uses maximum likelihood estimations based on the expectation–maximization algorithm to identify putative hybrids between two parental populations.

## 3 RESULTS

### 3.1 Whole genome sequencing

After removing low‐quality reads, 1.1 billion reads were retained across all samples (Table S1). The *Phymatolithon calcareum* reference assembled using reads from Mor02 was 68 Mbp in length, with 30X mean coverage, a contig N50 of 4371 bp and a GC content of 47.6%. Across all samples, 621 million reads mapped to the reference, although the number of mapped reads from *P. purpureum* and *Lithothamnion corallioides* individuals was very low (<5%). Three individuals of *P. calcareum* (Fal03, Mor10 and Zar09) were discarded as they had less than 20 high‐quality reads and zero alignments to the reference.

### 3.2 *Phymatolithon calcareum*—nuclear SNPs

The nuclear SNP data set for *P. calcareum* after filtering contained 71 individuals genotyped at 14,150 biallelic SNPs. No outliers were detected in this data set using OutFLANK. In addition, no individuals were removed due to clonality because the distribution of Prevosti genetic distances did not provide clear evidence of clones in the data set, though a few pairwise distances were close to zero (Figure S1). The Fal Estuary had by far the highest number of private alleles at 9299, while Illa de Ons had 1063 and the remaining sites either had zero or less than 100 private alleles (Figure S1). Allelic richness, on the other hand, was similar across all sites, but was highest in the Fal Estuary. Patterns of observed heterozygosity and *F*_IS_ were similar across all sites (Figure S1–S2), except for the Fal Estuary which had higher mean heterozygosity and far more loci with highly negative values of *F*_IS_ indicative of an excess of heterozygotes relative to Hardy–Weinberg expectations.

**FIGURE 1:**
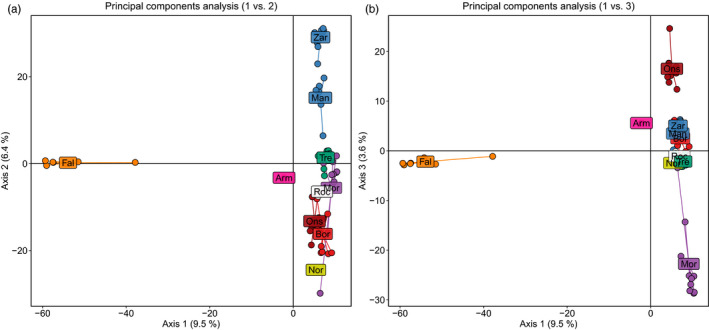
Principal component analysis (PCA) using the allele frequencies of 14,150 single nucleotide polymorphisms genotyped in 71 individuals. (a) PCA using axes 1 and 2; (b) PCA using axes 1 and 3. Colours denote site or geographical region of origin: Norway (yellow), Northern Ireland and south‐west England (blue), Fal Estuary (orange), Morlaix (purple), Trévignon (turquoise), La Rochelle (white), Bornalle (red), Illa de Ons (dark red) and Portugal (pink). In both PCAs, the position of the site labels represents the centroid mean of all the individuals from the site; see Table 1 for site code translation

Across all pairwise *F*_ST_ comparisons, the Fal Estuary was the most differentiated site, while the lowest *F*_ST_ value was between Zara Shoal and The Manacles (Figure S3). The PCA showed similar patterns to the *F*_ST_ heatmap, with strong separation between the Fal Estuary and all other samples on the first axis (Figure 1a). In addition, finer site‐level structure was detected within the main cluster of samples on the second axis, which was generally ordered by geography starting from the more northerly sites, Zara Shoal (Northern Ireland) and The Manacles (south‐west England), to the more southerly sites including Bornalle and Illa de Ons (north‐west Spain). However, the single samples from Norway (Nor01) and Portugal (Arm01), and a single individual from Morlaix (Mor05), did not conform to this geographical pattern. The third axis showed most of Morlaix to be differentiated from the other sites, and also revealed differentiation between Bornalle and Illa de Ons (Figure 1b).

**FIGURE 2:**
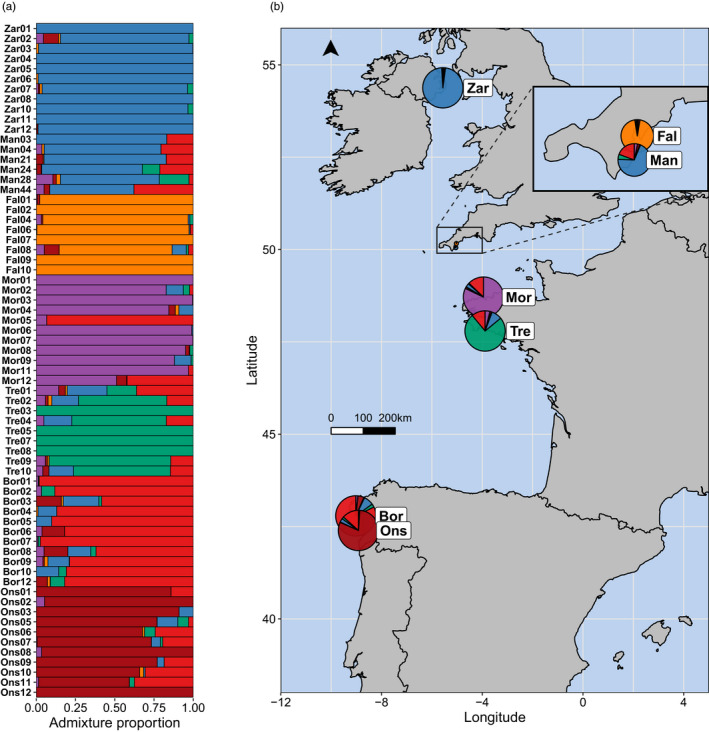
Admixture coefficients estimated using sparse non‐negative matrix factorization assuming *K* = 6, implemented using the R package LEA. (a) Individual admixture coefficients; each bar represents an individual and the colours denote admixture proportions to each *K* cluster (ancestral population). (b) Mean admixture proportions for each site; data are presented using pie charts and visualized on a map of western Europe showing the geographical location of each site. Some sampling sites from Table 1 are not present on the map because they either did not meet the filtering thresholds or had insufficient sample sizes for this analysis

In the SNMF analysis, the lowest cross‐entropy was observed when assuming four ancestral populations (Figure S4a). However, admixture results for *K*2‐7 were visualized (Figure S4b) and *K* = 6 revealed finer scale structure, which was supported by PCA results, so *K* = 6 was used for interpretation. This analysis indicated that the seven sites included in the SNMF model were organized into six main clusters: (i) Zara Shoal and The Manacles, (ii) the Fal Estuary, (iii) Morlaix, (iv) Trévignon, (v) Bornalle and (vi) Illa de Ons (Figure 2a,b). There was, however, evidence of admixture among these groups, although admixture was virtually absent in the Fal Estuary. Furthermore, as shown in the PCAs (Figure 1), Mor05 was markedly different from the other samples in Morlaix—this individual instead shared most of its ancestry with Bornalle (Figure 2a).

**FIGURE 3:**
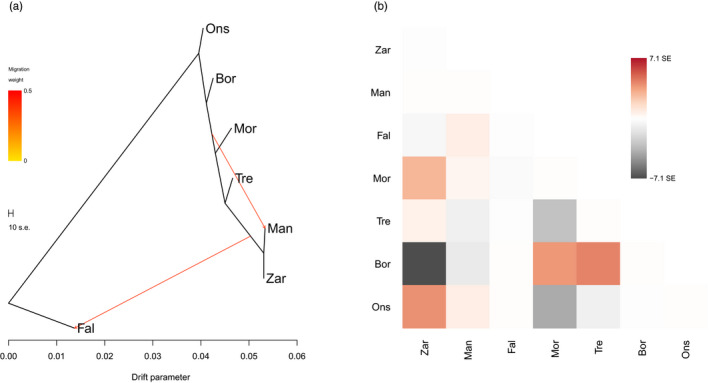
TreeMix results with two migration events modelled. (a) Maximum likelihood tree rooted with the Fal Estuary. (b) Residual fit of the observed versus the predicted squared allele frequency differences. Residuals above zero represent sites that are more closely related to each other in the data than in the tree

TreeMix results showed that modelling two migration events explained 98.4% of the variation in the data with no notable increase as more migration events were added (Figure S5); therefore, the tree generated using this model was used for interpretation. The genetic relationships in this tree (Figure 3a) showed very similar patterns to the PCA and the SNMF analysis, that is, the Fal Estuary was genetically divergent from the other sites and the main cluster of sites was separated geographically, the closest genetic relationship of which was between The Manacles and Zara Shoal. The two historic migration events inferred by the model suggested (i) gene flow from a population related to The Manacles/Zara Shoal to the Fal Estuary and (ii) gene flow from a population related to Morlaix to The Manacles (the source population of these events could be the aforementioned sites or a related population not sampled here). The residuals of the model were also visualized (Figure 3b) because residuals above zero can indicate pairs of sites where the model underestimates the observed covariance, which suggests that these sites are more closely related to each other in the data than in the best‐fit tree (Pickrell & Pritchard, 2012). This revealed that Bornalle is more closely related to both Morlaix and Trévignon in the data than represented in the tree, a result consistent with the SNMF analysis whereby similar patterns of admixture were observed.

### 3.3 *Phymatolithon calcareum*—organelle CDS

Across all *P. calcareum* samples, 69 mitogenomes and 75 plastomes were extracted (Table S2). For all plastomes, a circular and complete contig was assembled by Unicycler; however, assembling a circular and complete mitogenome was only possible for one individual, Tre04. For each individual, 24 mitochondrial coding sequences (CDS) and 213–214 plastid CDS were predicted by the GeSeq program. For instance, for Tre04, the complete mitogenome was 28,066 bp long and 24 CDS were annotated, while the complete plastome was 186,244 bp long and 214 CDs were annotated (Figure S6a,b). The concatenated mitogenome alignment contained 24 CDS and was 16,542 bp long; across this alignment, there were 32 variant sites (99.8% of all sites were invariant). The concatenated plastome alignment, on the other hand, contained 212 CDS and was 152,584 bp long; across this alignment, there were 417 variant sites (99.7% of all sites were invariant). Compared to the total sequence length of the mitogenome and plastome assemblies (Table S2), both concatenated alignments were much shorter in length. These shorter alignments were probably linked to the presence of redundant noncoding sequences in the assemblies that were not considered in the CDS alignments, and additionally for the plastome alignment, a failure to retrieve some CDS genes in some individuals. Hierarchical clustering showed that, overall, there are very few genetic differences among *P. calcareum* samples at both mitochondrial CDS and plastid CDS (Figure S7). Individuals from the same geographical sampling site tended to share more identical sites in CDS regions with each other or have completely identical mitogenome/plastome CDS. However, although Mor05 was identical to most other Morlaix samples in mitochondrial CDS (Figure S7a), Mor05 was separated from the main group of Morlaix samples in plastid CDS (Figure S7b), a result similar to the pattern found with nuclear SNPs.

### 3.4 *Phymatolithon purpureum* and *Lithothamnion corallioides*—organelle CDS

For *P. purpureum*, seven mitogenomes and nine plastomes were assembled. Of these, all mitogenome assemblies were linear and incomplete, while six (out of nine) plastome assemblies were circular and complete (Table S2). In comparison, for *L. corallioides*, two mitogenomes were assembled, of which one was circular and complete (Lcor09), while four plastomes were assembled, of which three were circular and complete (Table S2). The number of plastid CDS predicted in *P. purpureum* and *L. corallioides* was the same as for *P. calcareum* (between 213 and 214). In contrast, 23 CDS were predicted for *P. purpureum* mitogenomes, while 25 CDS were predicted for *L. corallioides* mitogenomes (Table S2; Figure S6c–f). BLASTn results of *cox1* and *psbA* CDS confirmed that all sequences corresponded to their expected species identification (Table S2). Hierarchical clustering using a concatenated mitogenome (23 CDS) and plastome (212 CDS) alignment showed clear separation at a species level (Figure S8). Additionally, the dendrograms revealed that *P. calcareum* shares more identical sites in CDS regions with *L. corallioides* than with *P. purpureum*.

### 3.5 Introgression analysis

**FIGURE 4:**
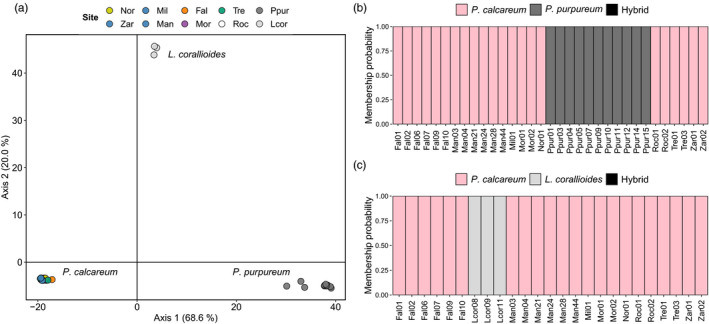
Analyses to test for the presence of introgression from *Phymatolithon purpureum* or *Lithothamnion corallioides* into *Phymatolithon calcareum* from the Fal Estuary. (a) Principal component analysis using the allele frequencies of 1130 single nucleotide polymorphisms genotyped in 36 individuals; see Table 1 for site code translation. On the right, membership probabilities for the *snapclust* hybrid tests are shown using (b) *P. purpureum* and (c) *L. corallioides*—absence of the hybrid membership (black) indicated that no hybrids were detected in these analyses

After filtering, the final SNP data set for testing introgression contained 1130 SNPs genotyped at 36 individuals. In comparison to the *P. calcareum* data set, the lower number of SNPs retained likely reflects the low number of alignments of *P. purpureum* and *L. corallioides* individuals to the reference, which could be due to a lack of quality sequence data for these samples (extracting sufficient quantities of DNA for whole genome sequencing is extremely challenging in maerl) and/or evolutionary divergence. Nevertheless, out of these SNP loci, 803 loci had no missing data so at least this number of loci was genotyped across all three species. A PCA using these SNPs showed clear separation at a species level, with Fal Estuary individuals all grouping with *P. calcareum* (Figure 4a). The introgression analyses using *snapclust* showed that no Fal Estuary individual, or indeed any *P. calcareum* individual, had any membership of the hybrid cluster with neither *P. purpureum* (Figure 4b) nor *L. corallioides* (Figure 4c).

## 4 DISCUSSION

In this study, we used a whole genome genotyping approach to generate, to our knowledge, the first population genomic data for a maerl‐forming species. We assembled a draft *Phymatolithon calcareum* reference from Illumina paired‐end data and genotyped samples at genome‐wide SNPs to analyse population genetic structure. Overall, our results indicate that *P. calcareum* maerl beds across the north‐east Atlantic are geographically structured and generally show a north‐south clinal pattern of genetic variation, a pattern similar to other benthic marine species distributed across a similar geographical area, including pink sea fans (Holland et al., 2017), European lobsters (Jenkins et al., 2019) and brown macroalgae (Neiva et al., 2014), and likely explained by the low dispersal capacity of *P*. *calcareum* and isolation‐by‐distance. In addition, we found that samples from the Fal Estuary are genetically distinct from all other sampling sites, further analysis of which showed that this pattern of divergence is not the result of introgression from *P. purpureum* or *L. corallioides*—two closely related species that are co‐distributed across the geographical range of *P. calcareum* (Pardo et al., 2014).

### 4.1 Fal Estuary maerl bed

**FIGURE 5:**
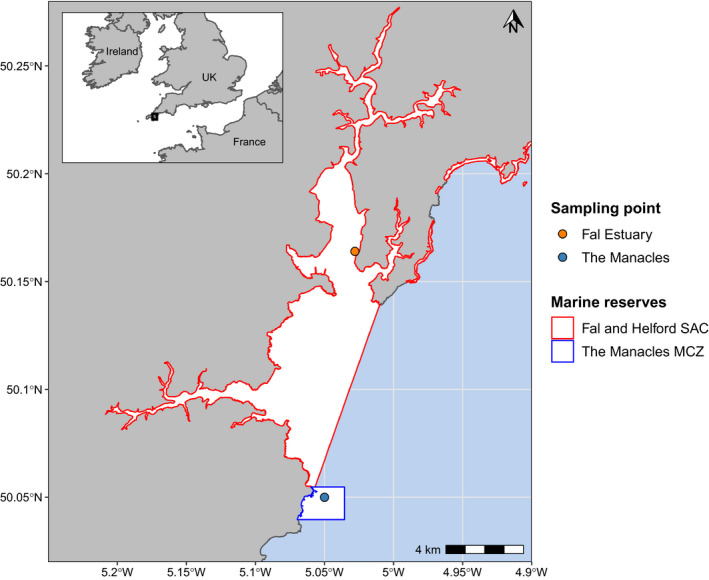
Map showing the sampling locations of *Phymatolithon calcareum* from the Fal Estuary and The Manacles, the former being a sheltered inner estuary site and the latter being an exposed outer estuary site. The boundaries of the Fal and Helford Special Area of Conservation (SAC) and The Manacles Marine Conservation Zone (MCZ) are shown

Distinct genetic diversity was detected in *P. calcareum* individuals from the Fal Estuary (Figures 1, 2, 3). Surprisingly, the Fal Estuary was even differentiated from individuals sampled from The Manacles, a site located only 13 km south of the Fal Estuary (Figure 5), but one that extends 0.7 km from the shoreline and is exposed to moderate tidal currents compared to the more sheltered Fal Estuary. One possible explanation for this distinct diversity, therefore, is that *P. calcareum* in the Fal Estuary are extremely isolated and have virtually no, or extremely rare, gene flow with *P. calcareum* from The Manacles and the rest of the Atlantic. Moreover, as coralline algae are extremely slow‐growing and their lifespans can reach hundreds of years (Foster, 2001; Goldberg, 2006), this suggests that this distinct diversity has likely arisen over a very long time frame. A similar pattern of genetic differentiation between inner and outer zones of estuaries was discovered among maerl beds in north‐west Spain in a previous study (Pardo et al., 2019), two sites of which are included in the present study—Bornalle (inner) and Illa de Ons (outer)—and our results using genome‐wide SNP markers (Figures 1 and 2) support the differentiation found previously between these two sites using microsatellite loci. Together, these results support the hypothesis that geographical isolation has contributed towards the distinct diversity of *P. calcareum* in the Fal Estuary. This may indicate that other maerl beds located within sheltered inner estuaries and inlets may also exhibit similar patterns of geographical isolation and harbour distinct genetic diversity. Accordingly, we recommend further research to explore whether maerl‐forming species within other estuaries (e.g. Helford River, England), sea lochs (e.g. western Scotland) and fjords (e.g. Norway) also exhibit these characteristics.

In this study, we also tested the hypothesis that individuals from the Fal Estuary are putative hybrids produced by introgression from a closely related species, as postulated by Pardo et al. (2019). A PCA showed that Fal Estuary samples grouped with *P. calcareum* and not with *P*.* purpureum* or *L. corallioides* (Figure 4a), and the hybrid tests using *snapclust* (Figure 4b,c) showed that no individuals from the Fal Estuary were hybrids; we can therefore reject the hypothesis of introgression from either of these two species. These results suggest that despite being distinct from other north‐east Atlantic populations of *P*. *calcareum*, the intra‐species divergence observed in the Fal Estuary is much less than the level of inter‐species divergence. However, because the hybrid tests were conducted using data for only *P*.* purpureum* and *L. corallioides*, we cannot rule out the possibility that introgression has occurred from another unsampled species of *Phymatolithon*, such as *P. lamii*, which is phylogenetically close to *P. calcareum* (Peña et al., 2020) and has been reported to exist in Brittany, north‐west France (Pardo et al., 2017). Similarly, although we sampled across the range of *P. calcareum*, there is still a possibility that the Fal Estuary population was introduced from an unsampled population of *P. calcareum*. Another potential explanation for the distinct diversity observed in the Fal Estuary is autopolyploidy. In their study, Pardo et al. (2019) reported that all *P. calcareum* samples collected from the Fal Estuary are possibly triploid sporophytes because samples displayed a triploid profile in three out of the eight microsatellite loci scored (PC‐2, PC‐3 and PC‐7). Interestingly, all Fal Estuary samples in the microsatellite study of Pardo et al. (2019) were also reported to be clones, whereas in our study using genome‐wide SNPs, the same samples did not show clear patterns of clonality. For instance, when analysing only polymorphic SNPs among Fal Estuary individuals that had no missing data (*N* = 6191 SNPs), the mean number of allelic differences among individuals was 2320 (range: 1738–2796). We did, however, find higher mean heterozygosity and more loci with negative *F*_IS_ values in the Fal Estuary, which could be interpreted as a signature of asexual reproduction, thereby supporting a hypothesis of clonality in this population. At present, autopolyploidy cannot be ruled as a potential explanation because we do not yet know the true ploidy of the Fal Estuary samples, but this could be explored in future research by estimating the genome size and ploidy of individuals using flow cytometry techniques.

In the UK and Ireland, both *P. calcareum* and *L. corallioides* are listed in Annex V of the EU Habitats Directive and the maerl beds they form are listed as a key habitat within the Annex I habitat definitions, ‘*large shallow inlet and bays*’ and ‘*sand banks which are slightly covered by seawater at all times*’ (OSPAR Commission, 2010). The Fal Estuary contains the largest maerl bed in England, composed of dense aggregations of both live and dead *P*. *calcareum* and *L. corallioides* (Allen et al., 2014), and harbours an exceptionally diverse biological community (Natural England, 2000). Currently, this maerl bed is situated within the Fal and Helford Special Area of Conservation (SAC), a protected area established to safeguard key European habitats listed in the Habitats Directive. The Fal and Helford SAC prohibits bottom trawling, but some other resource extraction and activities are still permitted (Natural England, 2000), such as the maintenance of a navigable waterway into Falmouth dock, a busy commercial and naval harbour. Although damage from trawling and dredging is mitigated by this by‐law, it is nevertheless important to consider indirect stressors that could compromise the integrity and health of the Fal Estuary maerl bed, such as increased sedimentation and pollution (Barbera et al., 2003) from boat traffic and sewage. Data from our study suggest that this site also harbours unique genetic diversity in *P. calcareum* which has likely developed over a long period of time; these results provide further evidence to support the current conservation management objectives of this SAC to ‘maintain’ the maerl bed in a ‘favourable condition’ (Natural England, 2000).

In comparison to the Fal Estuary, the nearby Manacles maerl bed has a relatively restricted distribution which is comprised primarily of dead maerl (Downie et al., 2018). This bed is situated within The Manacles Marine Conservation Zone (MCZ), a Marine Protected Area (MPA) established under the Marine and Coastal Access Act 2009, one of its aims of which is to ‘recover’ the maerl bed to a ‘favourable condition’. The characterization report of this MCZ cited that more data are needed to fully evaluate the condition and value of this maerl bed, one point of which outlined the need to investigate ‘*the composition and condition of the maerl species population of which the maerl beds are comprised’* (Downie et al., 2018). Our study serves to elucidate part of this knowledge gap—we have found that all maerl sampled from The Manacles were *P. calcareum* and that The Manacles population is genetically differentiated from the Fal Estuary population. Instead, admixture analyses suggest that The Manacles shares ancestry with Zara Shoal (Strangford Lough, Northern Ireland) or possibly from an unsampled source population related to Zara Shoal. Therefore, conservation management within England should consider *P*. *calcareum* maerl beds from the Fal Estuary and The Manacles as separate and distinct populations.

### 4.2 Morlaix and Trévignon maerl beds

In north‐west France, we detected genetic differentiation between Morlaix and Trévignon (Figures 1b and 2), two sites located on the northern and southern coast of Brittany, respectively, which are separated by approximately 190 km of coastal sea. Previous phylogeographic analysis of a polychaete worm revealed a distinct clade of *Owenia fusiformis* in Morlaix compared to other sites sampled around western and southern Brittany (Jolly et al., 2006). The authors reported that this clade might be composed of small remnant populations that may have persisted after the Last Glacial Maximum. Indeed, unique genetic diversity has been found in the Brittany region in a number of seaweed species (Hoarau et al., 2007; Neiva et al., 2014; Olsen et al., 2010), a finding often linked to glacial refugia (Maggs et al., 2008; Jenkins et al., 2018). Therefore, the genetic differentiation observed between Morlaix and Trévignon in the present study could be because of the existence of remnant populations of *P. calcareum*, which have persisted since the Last Glacial Maximum. Alternatively, the effects of topography and oceanography around the Brittany region (Dauvin, 2012; Nicolle et al., 2015), such as gyres and tides, could act to reduce connectivity with adjacent and distant maerl beds, which would also generate a similar pattern of genetic divergence. At present, two European MPAs have been designated under the Habitats Directive which include maerl beds from Morlaix (Baie de Morlaix MPA) and Trévignon (Dunes et côtes de Trévignon MPA). However, there are currently no known restrictions to bottom trawling in these MPAs (mpatlas.org/zones), the establishment of which is recommended to mitigate the risk of losing genetic and species diversity in these maerl bed habitats.

Interestingly, one individual from Morlaix, Mor05, was found to be differentiated from the main group of Morlaix samples at both nuclear and plastid DNA (Figures 1 and 2, Figure S7b). Instead, it shared most of its nuclear ancestry with maerl from Bornalle (north‐west Spain). These results suggest that Mor05 is likely a historic immigrant from a population in north‐west Spain. However, given the low dispersal potential of *P. calcareum* and that the geographical distance from Bornalle to Morlaix across the Bay of Biscay is over 600 km, it is unclear whether this was mediated by a single event of long‐distance dispersal, by multiple events of dispersal via stepping stone populations such as La Rochelle and Trévignon, or by some other mechanism such as human‐mediated translocation. A comparable pattern of genetic similarity was found between The Manacles (south‐west England) and Zara Shoal (Northern Ireland), a spatial scale similar to Bornalle and Morlaix, which may suggest that *P*. *calcareum* is indeed able to take advantage of infrequent events of extreme weather and ocean dynamics to disperse its gametes, spores or clones to distant environments (Pardo et al., 2019).

### 4.3 Loch Sween maerl bed

Loch Sween is a sea loch located in western Scotland which has been reported to contain two maerl beds, one of which was reported to be primarily composed of *P. calcareum* while the other was reported to be primarily composed of *Lithothamnion glaciale* (Moore et al., 2013). Although we attempted to sample *P. calcareum* from Loch Sween, identifying samples of maerl collected in the field to species level based on morphology is still extremely difficult, and our molecular analysis of organelle genomes suggested that these DNA samples were actually *P. purpureum*. Nevertheless, this finding suggests that there is a third species composing maerl beds in Loch Sween, which contrasts with the commissioned report detailing only two maerl‐forming species in the Loch Sween MPA (Moore et al., 2013). Therefore, although the majority of maerl from this bed in Loch Sween could indeed be *P. calcareum*, our results suggest that *P. purpureum* may be present in higher abundance than previously thought, and thus, we recommend that further research should be conducted on this maerl bed to verify species diversity and density.

Our hierarchical clustering analysis of mitochondrial and plastid CDS (Figure S8) also indicated that *P. purpureum* shares fewer organelle coding sequences with *P. calcareum* than *L. corallioides* does with *P. calcareum*. Originally, *P. purpureum* was described as *Lithothamnion purpureum* but the genus was changed to *Phymatolithon*, and a recent study using *P. purpureum* samples from France, Ireland and the UK appeared to validate this change in genus via morphological and molecular analysis (Jeong et al., 2019). However, our analysis suggests that the taxonomy may not be completely resolved, at least for *P. purpureum* from Loch Sween, and that the classification of *P. purpureum* into the genus *Phymatolithon* may need to be re‐evaluated.

### 4.4 Conclusion

In conclusion, the results of our whole genome genotyping study reinforce management guidelines advocated in previous reports on maerl‐forming species, that is, the genetic and species diversity of maerl bed ecosystems has likely accumulated over a large temporal scale and that maerl beds in western Europe should be considered a nonrenewable resource (Barbera et al., 2003; Foster, 2001; OSPAR Commission, 2010; Pardo et al., 2019). Moreover, the increased resolution afforded by using genome‐wide SNPs suggests that genomic diversity in *P. calcareum* is partitioned across a range of spatial scales, likely driven by low dispersal capacity, asexual reproduction and localized isolation. This is particularly apparent in the Fal Estuary, in which distinct genetic diversity was found in *P. calcareum* relative to the other sites sampled in our study. Crucially, both genetic and species diversity are important for maximizing ecosystem resilience and adaptation to climate and environmental change (Garner et al., 2020; Laikre et al., 2020). Our results emphasize that *P. calcareum* maerl beds should be (or continue to be) managed and monitored as independent units across the north‐east Atlantic.

## CONFLICT OF INTEREST

The authors declare that there is no conflict of interest.

## Supporting information

Figures S1–S8

Table S1

Table S2

## Data Availability

Raw DNA sequence data are available from the NCBI (BioProject: PRJNA682082; SRA: SRX9781154–SRX9781248). Supplementary data, organelle genomes and code are available from GitHub (https://github.com/Tom‐Jenkins/maerl_genomics).

## References

[eva13219-bib-0001] Adey, W. H. , & McKibbin, D. L. (1970). Studies on the maerl species *Phymatolithon calcareum* (Pallas) nov. comb. and *Lithothamnium coralloides* Crouan in the Ría de Vigo. Botanica Marina, XIII, 100–106.

[eva13219-bib-0002] Allen, C. , Axelsson, M. , Dewey, S. , & Wilson, J. (2014). Fal and Helford SAC Maerl Drop‐down Video and Dive Survey 2013. A report to Natural England by Seastar Survey Ltd. 1–89. Available online at: https://bit.ly/393iQ3H

[eva13219-bib-0003] Barbera, C. , Bordehore, C. , Borg, J. A. , Glémarec, M. , Grall, J. , Hall‐Spencer, J. M. , DeLaHuz, C. H. , Lanfranco, E. , Lastra, M. , Moore, P. G. , & Mora, J. (2003). Conservation and management of northeast Atlantic and Mediterranean maerl beds. Aquatic Conservation: Marine and Freshwater Ecosystems, 13, 65–76.

[eva13219-bib-0004] Bernard, G. , Romero‐Ramirez, A. , Tauran, A. , Pantalos, M. , Deflandre, B. , Grall, J. , & Grémare, A. (2019). Declining maerl vitality and habitat complexity across a dredging gradient: insights from in situ sediment profile imagery (SPI). Scientific Reports, 9, 16463. 31712682 10.1038/s41598-019-52586-8 PMC6848171

[eva13219-bib-0005] Beugin, M. P. , Gayet, T. , Pontier, D. , Devillard, S. , & Jombart, T. (2018). A fast likelihood solution to the genetic clustering problem. Methods in Ecology and Evolution, 9, 1006–1016. 29938015 10.1111/2041-210X.12968 PMC5993310

[eva13219-bib-0006] Blake, C. , & Maggs, C. A. (2003). Comparative growth rates and internal banding periodicity of maerl species (Corallinales, Rhodophyta) from northern Europe. Phycologia, 42, 606–612.

[eva13219-bib-0007] Brodie, J. , Williamson, C. J. , Smale, D. A. , Kamenos, N. A. , Mieszkowska, N. , Santos, R. , Cunliffe, M. , Steinke, M. , Yesson, C. , Anderson, K. M. , & Asnaghi, V. (2014). The future of the northeast Atlantic benthic flora in a high CO_2_ world. Ecology and Evolution, 4, 2787–2798. 25077027 10.1002/ece3.1105 PMC4113300

[eva13219-bib-0008] Carro, B. , Lopez, L. , Peña, V. , Bárbara, I. , & Barreiro, R. (2014). DNA barcoding allows the accurate assessment of European maerl diversity: a proof‐of‐concept study. Phytotaxa, 190, 176–189.

[eva13219-bib-0009] Chen, S. , Zhou, Y. , Chen, Y. , & Gu, J. (2018). Fastp: an ultra‐fast all‐in‐one FASTQ preprocessor. Bioinformatics, 34, i884–i890. 30423086 10.1093/bioinformatics/bty560 PMC6129281

[eva13219-bib-0010] Collén, J. , Porcel, B. , Carré, W. , Ball, S. G. , Chaparro, C. , Tonon, T. , Barbeyron, T. , Michel, G. , Noel, B. , Valentin, K. , & Elias, M. (2013). Genome structure and metabolic features in the red seaweed *Chondrus crispus* shed light on evolution of the Archaeplastida. Proceedings of the National Academy of Sciences, 110, 5247–5252. 10.1073/pnas.1221259110 PMC3612618 23503846

[eva13219-bib-0011] Cornwall, C. E. , Diaz‐Pulido, G. , & Comeau, S. (2019). Impacts of ocean warming on coralline algae: knowledge gaps and key recommendations for future research. Frontiers in Marine Science, 6, 186.

[eva13219-bib-0012] Danecek, P. , Auton, A. , Abecasis, G. , Albers, C. A. , Banks, E. , DePristo, M. A. , Handsaker, R. E. , Lunter, G. , Marth, G. T. , Sherry, S. T. , & McVean, G. (2011). The variant call format and VCFtools. Bioinformatics, 27, 2156–2158. 21653522 10.1093/bioinformatics/btr330 PMC3137218

[eva13219-bib-0013] Dauvin, J.‐C. (2012). Are the eastern and western basins of the English Channel two separate ecosystems? Marine Pollution Bulletin, 64, 463–471. 22245434 10.1016/j.marpolbul.2011.12.010

[eva13219-bib-0014] Downie, A. , Eggleton, J. , McIlwaine, P. , & Bullimore, R. (2018). The Manacles Marine Conservation Zone (MCZ) Characterisation Report 2015. MPA Monitoring Programme. Contract Reference: MB0129, Report Number: 2, Version: 4. Available online at: https://bit.ly/2QnAA38

[eva13219-bib-0015] Foster, M. S. (2001). Rhodoliths: between rocks and soft places. Journal of Phycology, 37, 659–667.

[eva13219-bib-0016] Frichot, E. , & François, O. (2015). LEA: an R package for landscape and ecological association studies. Methods in Ecology and Evolution, 6, 925–929.

[eva13219-bib-0017] Funk, W. C. , McKay, J. K. , Hohenlohe, P. A. , & Allendorf, F. W. (2012). Harnessing genomics for delineating conservation units. Trends in Ecology & Evolution, 27, 489–496. 22727017 10.1016/j.tree.2012.05.012 PMC4185076

[eva13219-bib-0018] Garner, B. A. , Hoban, S. , & Luikart, G. (2020). IUCN Red List and the value of integrating genetics. Conservation Genetics, 21, 795–801.

[eva13219-bib-0019] Goldberg, N. (2006). Age estimates and description of rhodoliths from Esperance Bay, Western Australia. Journal of the Marine Biological Association of the United Kingdom, 86, 1291–1296.

[eva13219-bib-0020] Goudet J. (2005). hierfstat, a package for r to compute and test hierarchical F‐statistics. Molecular Ecology Notes, 5(1), 184–186. 10.1111/j.1471-8286.2004.00828.x

[eva13219-bib-0021] Hall‐Spencer, J. M. , Grall, J. , Moore, P. G. , & Atkinson, R. J. A. (2003). Bivalve fishing and maerl‐bed conservation in France and the UK – retrospect and prospect. Aquatic Conservation: Marine and Freshwater Ecosystems, 13, S33–S41.

[eva13219-bib-0022] Hall‐Spencer, J. M. , & Moore, P. G. (2000). Scallop dredging has profound, long‐term impacts on maerl habitats. ICES Journal of Marine Science, 57, 1407–1415.

[eva13219-bib-0023] Hall‐Spencer, J. , White, N. , Gillespie, E. , Gillham, K. , & Foggo, A. (2006). Impact of fish farms on maerl beds in strongly tidal areas. Marine Ecology Progress Series, 326, 1–9.

[eva13219-bib-0024] Hernandez‐Kantun, J. , Hall‐Spencer, J. , Grall, J. , Adey, W. , Rindi, F. , Maggs, C. , Bárbara, I. , & Peña, V. (2017). North Atlantic rhodolith beds. In R. Riosmena‐Rodríguez , W. Nelson , & J. Aguirre (Eds.) Rhodolith/Maërl beds: A global perspective. Coastal Research Library (15, pp. 265–279). Cham: Springer.

[eva13219-bib-0025] Hoarau, G. , Coyer, J. A. , Veldsink, J. H. , Stam, W. T. , & Olsen, J. L. (2007). Glacial refugia and recolonization pathways in the brown seaweed *Fucus serratus* . Molecular Ecology, 16, 3606–3616. 17845434 10.1111/j.1365-294X.2007.03408.x

[eva13219-bib-0026] Holland, L. P. , Jenkins, T. L. , & Stevens, J. R. (2017). Contrasting patterns of population structure and gene flow facilitate exploration of connectivity in two widely distributed temperate octocorals. Heredity, 119, 35–48. 28295035 10.1038/hdy.2017.14 PMC5520136

[eva13219-bib-0027] Jackman, S. D. , Vandervalk, B. P. , Mohamadi, H. , Chu, J. , Yeo, S. , Hammond, S. A. , Jahesh, G. , Khan, H. , Coombe, L. , Warren, R. L. , & Birol, I. (2017). ABySS 2.0: resource‐efficient assembly of large genomes using a bloom filter effect of bloom filter false positive rate. Genome Research, 27, 768–777. 28232478 10.1101/gr.214346.116 PMC5411771

[eva13219-bib-0028] Jenkins, T. L. , Ellis, C. D. , & Stevens, J. R. (2018). SNP discovery in European lobster (Homarus gammarus ) using RAD sequencing. Conservation Genetics Resources, 11, 253–257. 10.1007/s12686-018-1001-8

[eva13219-bib-0029] Jenkins, T. L. , Ellis, C. D. , Triantafyllidis, A. , & Stevens, J. R. (2019). Single nucleotide polymorphisms reveal a genetic cline across the north‐east Atlantic and enable powerful population assignment in the European lobster. Evolutionary Applications, 12, 1881–1899. 31700533 10.1111/eva.12849 PMC6824076

[eva13219-bib-0030] Jenkins, T. L. , & Stevens, J. R. (2018). Assessing connectivity between MPAs: selecting taxa and translating genetic data to inform policy. Marine Policy, 94, 165–173.

[eva13219-bib-0031] Jeong, S. Y. , Won, B. Y. , Hassel, K. , & Cho, T. O. (2019). Revision of *Phymatolithon purpureum* (Hapalidiales, Rhodophyta) based on ultrastructural and molecular data. European Journal of Phycology, 54, 326–341.

[eva13219-bib-0032] Jin, J.‐J. , Yu, W.‐B. , Yang, J.‐B. , Song, Y. , DePamphilis, C. W. , Yi, T.‐S. , & Li, D.‐Z. (2020). GetOrganelle: a fast and versatile toolkit for accurate de novo assembly of organelle genomes. Genome Biology, 24(241). 10.1186/s13059-020-02154-5 PMC7488116 32912315

[eva13219-bib-0033] Jolly, M. T. , Viard, F. , Gentil, F. , Thiébaut, E. , & Jollivet, D. (2006). Comparative phylogeography of two coastal polychaete tubeworms in the Northeast Atlantic supports shared history and vicariant events. Molecular Ecology, 15, 1841–1855. 16689902 10.1111/j.1365-294X.2006.02910.x

[eva13219-bib-0034] Jombart, T. , & Ahmed, I. (2011). adegenet 1.3‐1: new tools for the analysis of genome‐wide SNP data. Bioinformatics, 27, 3070–3071. 21926124 10.1093/bioinformatics/btr521 PMC3198581

[eva13219-bib-0035] Kamenos, N. A. , Moore, P. G. , & Hall‐spencer, J. M. (2004). Nursery‐area function of maerl grounds for juvenile queen scallops. Marine Ecology Progress Series, 274, 183–189.

[eva13219-bib-0036] Kamenos, N. A. , Moore, P. G. , & Hall‐Spencer, J. M. (2004). Small‐scale distribution of juvenile gadoids in shallow inshore waters; what role does maerl play? ICES Journal of Marine Science, 61, 422–429.

[eva13219-bib-0037] Kamvar, Z. N. , Tabima, J. F. , & Grünwald, N. J. (2014). Poppr: an R package for genetic analysis of populations with clonal, partially clonal, and/or sexual reproduction. PeerJ, 2, e281. 24688859 10.7717/peerj.281 PMC3961149

[eva13219-bib-0038] Laikre, L. , Hoban, S. , Bruford, M. W. , Segelbacher, G. , Allendorf, F. W. , Gajardo, G. , Rodríguez, A. G. , Hedrick, P. W. , Heuertz, M. , Hohenlohe, P. A. , & Jaffé, R. (2020). Post‐2020 goals overlook genetic diversity. Science, 367, 1083–1085. 10.1126/science.abb2748 32139534

[eva13219-bib-0039] Li, H. (2011). A statistical framework for SNP calling, mutation discovery, association mapping and population genetical parameter estimation from sequencing data. Bioinformatics, 27, 2987–2993. 21903627 10.1093/bioinformatics/btr509 PMC3198575

[eva13219-bib-0040] Li, H. , Handsaker, B. , Wysoker, A. , Fennell, T. , Ruan, J. , Homer, N. , Marth, G. , Abecasis, G. , & Durbin, R. (2009). The Sequence Alignment/Map format and SAMtools. Bioinformatics, 25, 2078–2079. 19505943 10.1093/bioinformatics/btp352 PMC2723002

[eva13219-bib-0041] Maggs, C. A. , Castilho, R. , Foltz, D. , Henzler, C. , Jolly, M. T. , Kelly, J. , Olsen, J. , Perez, K. E. , Stam, W. , Väinölä, R. , Viard, F. , & Wares, J. (2008). Evaluating signatures of glacial refugia for North Atlantic benthic marine taxa. Ecology, 89, 108–122. 10.1890/08-0257.1 19097488

[eva13219-bib-0042] Mao, J. , Burdett, H. L. , McGill, R. A. R. , Newton, J. , Gulliver, P. , & Kamenos, N. A. (2020). Carbon burial over the last four millennia is regulated by both climatic and land use change. Global Change Biology, 26, 2496–2504. 10.1111/gcb.15021 32100446

[eva13219-bib-0043] Melbourne, L. A. , Hernández‐Kantún, J. J. , Russell, S. , & Brodie, J. (2017). There is more to maerl than meets the eye: DNA barcoding reveals a new species in Britain, *Lithothamnion erinaceum* sp. nov. (Hapalidiales, Rhodophyta). European Journal of Phycology, 52, 166–178.

[eva13219-bib-0044] Montecinos, A. E. , Guillemin, M. L. , Couceiro, L. , Peters, A. F. , Stoeckel, S. , & Valero, M. (2017). Hybridization between two cryptic filamentous brown seaweeds along the shore: analysing pre‐ and postzygotic barriers in populations of individuals with varying ploidy levels. Molecular Ecology, 26, 3497–3512. 28295812 10.1111/mec.14098

[eva13219-bib-0045] Moore, C. G. , Harries, D. B. , Atkinson, R. J. A. , Clark, L. , Cook, R. L. , Hirst, N. E. , Saunders, G. R. , Lyndon, A. R. , Sanderson, W. G. & Porter, J. S. (2013). The distribution and condition of proposed protected features within the Loch Sween possible Nature Conservation MPA. Scottish NatureScot Commissioned Report: No. 621, 1‐261. Available online at: https://bit.ly/3shWYJp

[eva13219-bib-0046] Murúa, P. , Edrada‐Ebel, R. A. , Muñoz, L. , Soldatou, S. , Legrave, N. , Müller, D. G. , Patiño, D. J. , van West, P. , Küpper, F. C. , Westermeier, R. , Ebel, R. , & Peters, A. F. (2020). Morphological, genotypic and metabolomic signatures confirm interfamilial hybridization between the ubiquitous kelps *Macrocystis* (Arthrothamnaceae) and *Lessonia* (Lessoniaceae). Scientific Reports, 10, 8279. 32427928 10.1038/s41598-020-65137-3 PMC7237481

[eva13219-bib-0047] Nakamura, Y. , Sasaki, N. , Kobayashi, M. , Ojima, N. , Yasuike, M. , Shigenobu, Y. , Satomi, M. , Fukuma, Y. , Shiwaku, K. , Tsujimoto, A. , & Kobayashi, T. (2013). The first symbiont‐free genome sequence of marine red alga, Susabi‐nori (*Pyropia yezoensis*). PLoS One, 8, e57122. 23536760 10.1371/journal.pone.0057122 PMC3594237

[eva13219-bib-0048] Natural England . (2000). Fal and Helford SAC Regulation Conservation Advice Package. UK0013112A.

[eva13219-bib-0049] Neiva, J. , Assis, J. , Fernandes, F. , Pearson, G. A. , & Serrão, E. A. (2014). Species distribution models and mitochondrial DNA phylogeography suggest an extensive biogeographical shift in the high‐intertidal seaweed *Pelvetia canaliculata* . Journal of Biogeography, 41, 1137–1148.

[eva13219-bib-0050] Nelson, W. A. (2009). Calcified macroalgae critical to coastal ecosystems and vulnerable to change: a review. Marine & Freshwater Research, 60, 787–801.

[eva13219-bib-0051] Nicolle, A. , Moitié, R. , Ogor, J. , Dumas, F. , Foveau, A. , Foucher, E. , & Thiébaut, E. (2015). Modelling larval dispersal of *Pecten maximus* in the English Channel: a tool for the spatial management of the stocks. ICES Journal of Marine Science, 74, 1812–1825.

[eva13219-bib-0052] Noisette, F. , Duong, G. , Six, C. , Davoult, D. , & Martin, S. (2013). Effects of elevated pCO_2_ on the metabolism of a temperate rhodolith *Lithothamnion corallioides* grown under different temperatures. Journal of Phycology, 49, 746–757. 27007207 10.1111/jpy.12085

[eva13219-bib-0053] Olsen, J. L. , Zechman, F. W. , Hoarau, G. , Coyer, J. A. , Stam, W. T. , Valero, M. , & Åberg, P. (2010). The phylogeographic architecture of the fucoid seaweed *Ascophyllum nodosum*: an intertidal ‘marine tree’ and survivor of more than one glacial‐interglacial cycle. Journal of Biogeography, 37, 842–856.

[eva13219-bib-0054] OSPAR Commission (2010). Background Document for Maërl Beds., ISBN, 978‐1‐907390‐32‐6.

[eva13219-bib-0055] Palsbøll, P. J. , Bérubé, M. , & Allendorf, F. W. (2007). Identification of management units using population genetic data. Trends in Ecology & Evolution, 22, 11–16. 16982114 10.1016/j.tree.2006.09.003

[eva13219-bib-0056] Pardo, C. , Bárbara, I. , Barreiro, R. , & Peña, V. (2017). Insights into species diversity of associated crustose coralline algae (Corallinophycidae, Rhodophyta) with Atlantic European maerl beds using DNA barcoding. An del Jard Bot Madrid, 74, e059.

[eva13219-bib-0057] Pardo, C. , Guillemin, M.‐L. , Peña, V. , Bárbara, I. , Valero, M. , & Barreiro, R. (2019). Local coastal configuration rather than latitudinal gradient shape clonal diversity and genetic structure of *Phymatolithon calcareum* maerl beds in North European Atlantic. Frontiers in Marine Science, 6, 149.

[eva13219-bib-0058] Pardo, C. , Lopez, L. , Peña, V. , Hernández‐Kantún, J. , Le Gall, L. , Bárbara, I. , & Barreiro, R. (2014). A multilocus species delimitation reveals a striking number of species of coralline algae forming maerl in the OSPAR maritime area. PLoS One, 9, e104073. 25111057 10.1371/journal.pone.0104073 PMC4128821

[eva13219-bib-0059] Peña, V. , Bárbara, I. , Grall, J. , Maggs, C. A. , & Hall‐Spencer, J. M. (2014). The diversity of seaweeds on maerl in the NE Atlantic. Marine Biodiversity, 44, 533–551.

[eva13219-bib-0060] Peña, V. , Vieira, C. , Braga, J. C. , Aguirre, J. , Rösler, A. , Baele, G. , De Clerck, O. , & Le Gall, L. (2020). Radiation of the coralline red algae (Corallinophycidae, Rhodophyta) crown group as inferred from a multilocus time‐calibrated phylogeny. Molecular Phylogenetics and Evolution, 150, 106845. 32360706 10.1016/j.ympev.2020.106845

[eva13219-bib-0061] Pickrell, J. K. , & Pritchard, J. K. (2012). Inference of population splits and mixtures from genome‐wide allele frequency data. PLoS Genetics, 8, e1002967. 23166502 10.1371/journal.pgen.1002967 PMC3499260

[eva13219-bib-0062] Pinho Costa, A. C. , Garcia, T. M. , Paiva, B. P. , Ximenes Neto, A. R. , & de Oliveira, S. M. (2020). Seagrass and rhodolith beds are important seascapes for the development of fish eggs and larvae in tropical coastal areas. Marine Environment Research, 161, 105064. 10.1016/j.marenvres.2020.105064 32784115

[eva13219-bib-0063] Qui‐Minet, Z. N. , Coudret, J. , Davoult, D. , Grall, J. , Mendez‐Sandin, M. , Cariou, T. , & Martin, S. (2019). Combined effects of global climate change and nutrient enrichment on the physiology of three temperate maerl species. Ecology and Evolution, 9, 13787–13807. 31938482 10.1002/ece3.5802 PMC6953553

[eva13219-bib-0064] R Core Team (2020). R: a language and environment for statistical computing. R Foundation for Statistical Computing. https://www.R‐project.org/.

[eva13219-bib-0065] Ragazzola, F. , Foster, L. C. , Jones, C. J. , Scott, T. B. , Fietzke, J. , Kilburn, M. R. , & Schmidt, D. N. (2016). Impact of high CO_2_ on the geochemistry of the coralline algae *Lithothamnion glaciale* . Scientific Reports, 6, 20572. 26853562 10.1038/srep20572 PMC4744931

[eva13219-bib-0066] Roach, M. J. , Schmidt, S. A. , & Borneman, A. R. (2018). Purge Haplotigs: allelic contig reassignment for third‐gen diploid genome assemblies. BMC Bioinformatics, 19, 460. 30497373 10.1186/s12859-018-2485-7 PMC6267036

[eva13219-bib-0067] Savoie, A. M. , & Saunders, G. W. (2015). Evidence for the introduction of the Asian red alga *Neosiphonia japonica* and its introgression with *Neosiphonia harveyi* (Ceramiales, Rhodophyta) in the Northwest Atlantic. Molecular Ecology, 24, 5927–5937. 26477438 10.1111/mec.13429

[eva13219-bib-0068] Sheehan, E. V. , Bridger, D. , & Attrill, M. J. (2015). The ecosystem service value of living versus dead biogenic reef. Estuarine, Coastal and Shelf Science, 154, 248–254.

[eva13219-bib-0069] Tamura, K. , & Nei, M. (1993). Estimation of the number of nucleotide substitutions in the control region of mitochondrial DNA in humans and chimpanzees. Molecular Biology and Evolution, 10, 512–526. 8336541 10.1093/oxfordjournals.molbev.a040023

[eva13219-bib-0070] Tillich, M. , Lehwark, P. , Pellizzer, T. , Ulbricht‐Jones, E. S. , Fischer, A. , Bock, R. , & Greiner, S. (2017). GeSeq ‐ versatile and accurate annotation of organelle genomes. Nucleic Acids Research, 45, W6–W11. 28486635 10.1093/nar/gkx391 PMC5570176

[eva13219-bib-0071] Van Der Heijden, L. H. , & Kamenos, N. A. (2015). Reviews and syntheses: calculating the global contribution of coralline algae to total carbon burial. Biogeosciences, 12, 6429–6441.

[eva13219-bib-0072] Vasimuddin, S. M. , Heng, L. , & Srinivas, A. (2019). Efficient architecture‐aware acceleration of BWA‐MEM for multicore systems. IEEE Parallel and Distributed Processing Symposium, 1‐17. https://arxiv.org/abs/1907.12931

[eva13219-bib-0073] Weir, B. S. , & Cockerham, C. C. (1984). Estimating *F*‐statistics for the analysis of population structure. Evolution, 38, 1358–1370. 28563791 10.1111/j.1558-5646.1984.tb05657.x

[eva13219-bib-0074] Whitlock, M. C. , & Lotterhos, K. E. (2015). Reliable detection of loci responsible for local adaptation: Inference of a null model through trimming the distribution of *F* _ST_ . American Naturalist, 186, S24–S36. 10.1086/682949 26656214

[eva13219-bib-0075] Wick, R. R. , Judd, L. M. , Gorrie, C. L. , & Holt, K. E. (2017). Unicycler: resolving bacterial genome assemblies from short and long sequencing reads. PLoS Computational Biology, 13, e1005595. 28594827 10.1371/journal.pcbi.1005595 PMC5481147

[eva13219-bib-0076] Willing, E. M. , Dreyer, C. , & van Oosterhout, C. (2012). Estimates of genetic differentiation measured by *F* _ST_ do not necessarily require large sample sizes when using many SNP markers. PLoS One, 7, e42649. 22905157 10.1371/journal.pone.0042649 PMC3419229

