# Supplementary material for: Whole genome genotyping reveals discrete genetic diversity in north‐east Atlantic maerl beds
Source: Evol Appl. 2021 Mar 30;14(6):1558–71. doi: 10.1111/eva.13219 (PMC8210795; doi:10.1111/eva.13219)
Supplement: Supplementary file 1 — Figures S1–S8 [file EVA-14-1558-s003.pdf]

Whole genome genotyping reveals discrete genetic diversity in north-east Atlantic maerl beds

Supplementary Figures

Jenkins et al. 2021

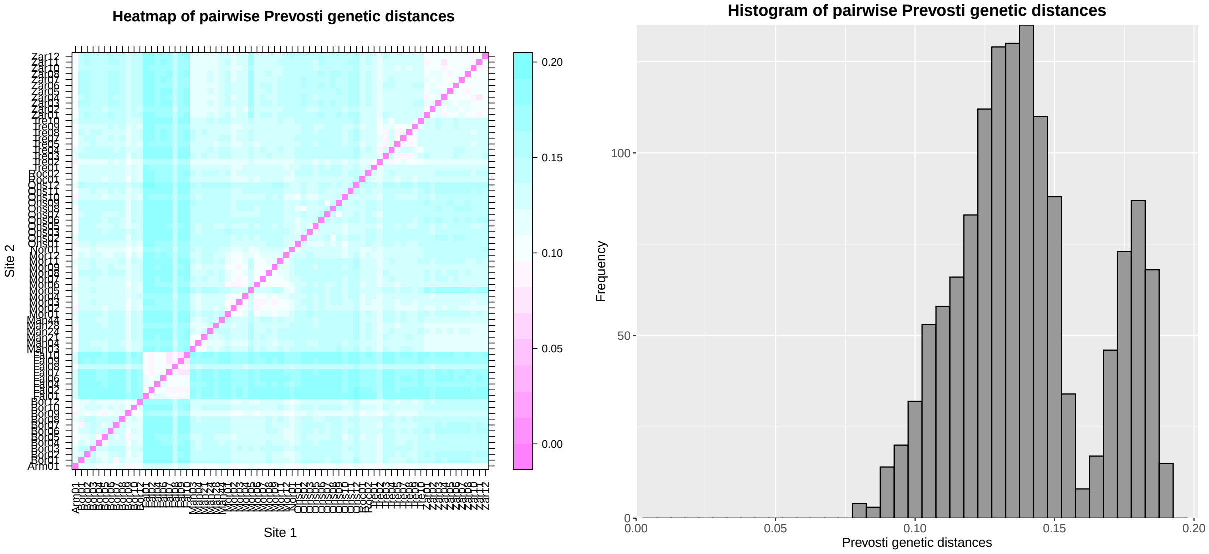

Site	Number of private alleles	Allelic richness	Mean observed heterozygosity	Mean F_{IS}
Armacao de Pera	0	NA	NA	NA
Bornalle	10	1.15	0.18	-0.13
Fal Estuary	9 299	1.18	0.25	-0.28
The Manacles	0	1.16	0.18	-0.12
Morlaix	74	1.14	0.17	-0.13
Norway	0	NA	NA	NA
Illa de Ons	1 063	1.16	0.19	-0.13
La Rochelle	0	1.16	NA	-0.13
Trevignon	46	1.15	0.18	-0.13
Zara Shoal	22	1.14	0.17	-0.16

Figure S1. Distribution of Prevosti genetic distances visualised with a heatmap (top-left) and a histogram (top-right). The heatmap shows that the highest pairwise genetic distances are between individuals from the Fal Estuary and all other individuals. The histogram shows no individuals with a pairwise distance of zero and that no clear gap is present between individuals of low genetic distances, the presence of which can be indicative of clones in the data set. The table shows the number of private alleles, allelic richness, and mean observed heterozygosity per SNP and mean F_{IS} per SNP at each site.

(a)

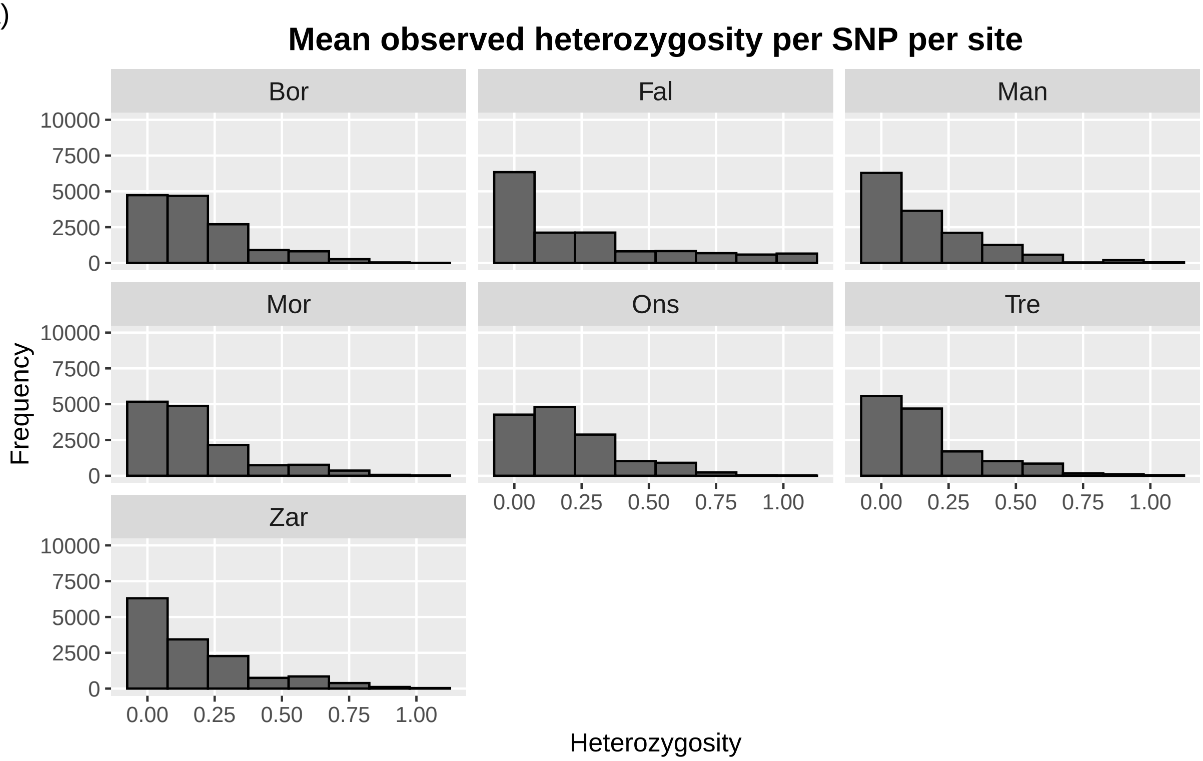

(b)

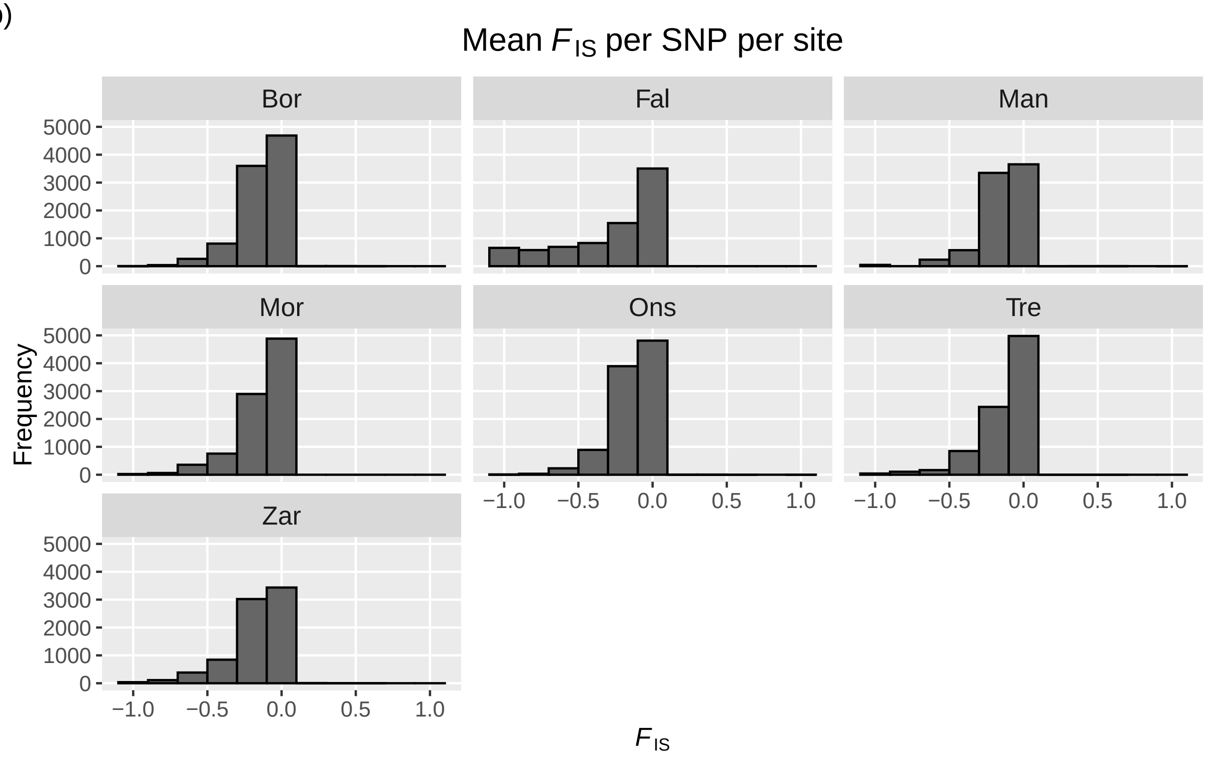

Figure S2. (a) Mean observed heterozygosity per SNP per site and (b) mean F_{IS} per SNP per site. Site codes are explained in Table 1.

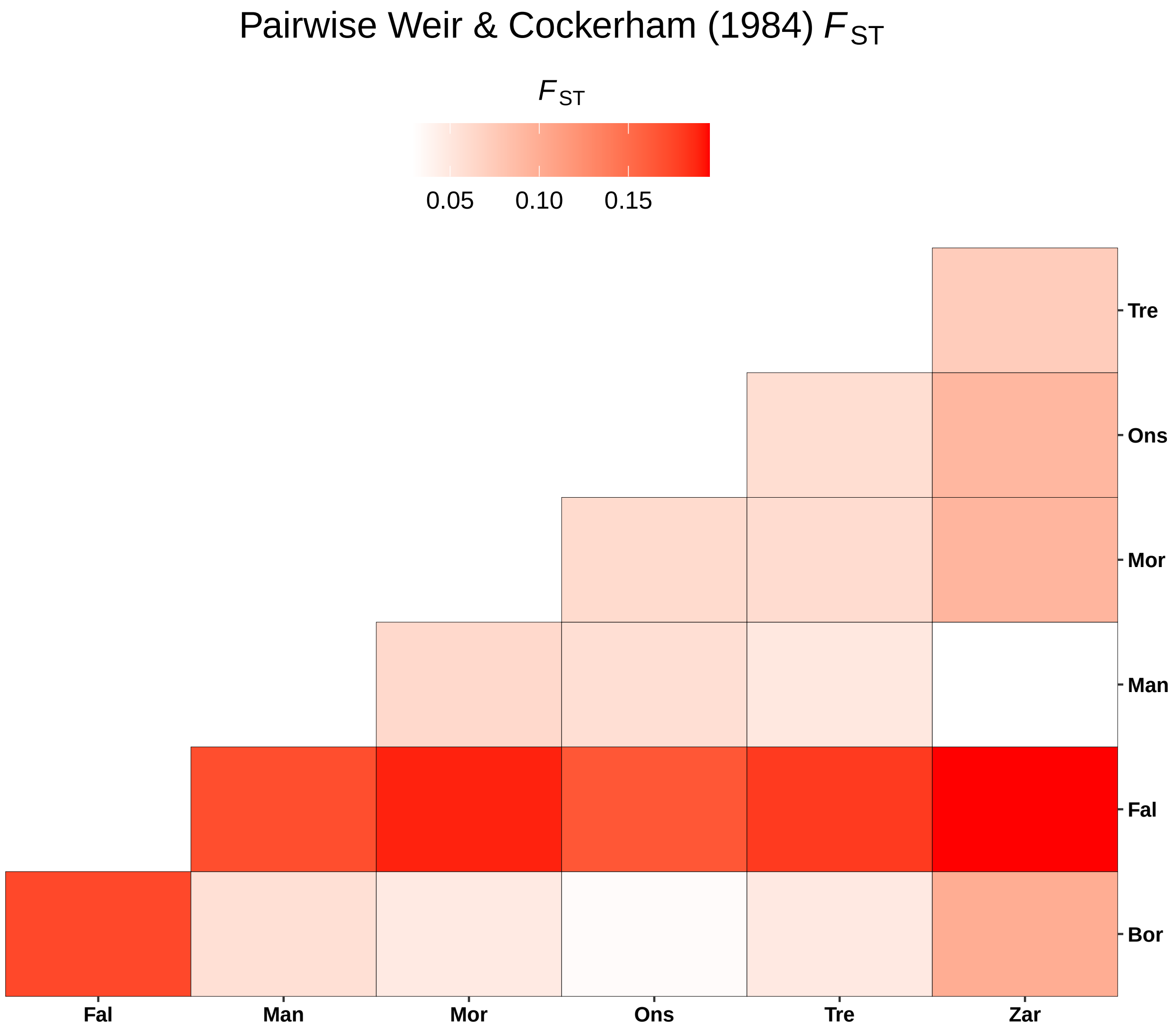

Figure S3. Heatmap of pairwise F_{ST} (Weir & Cockerham 1984). Site codes are explained in Table 1.

(a)

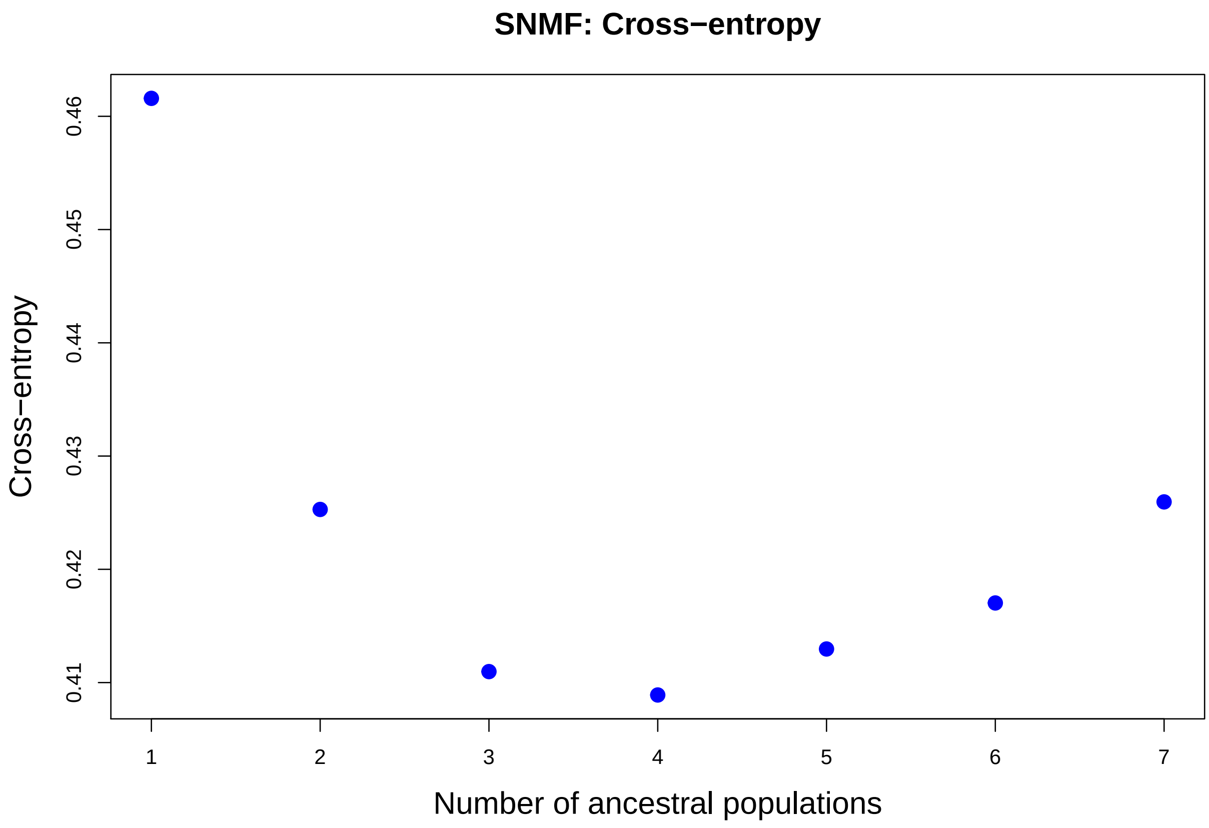
SNMF: Cross−entropy
Cross−entropy
0.41
0.42
0.43
0.44
0.45
0.46
1
2
3
4
5
6
7
Number of ancestral populations

(b)

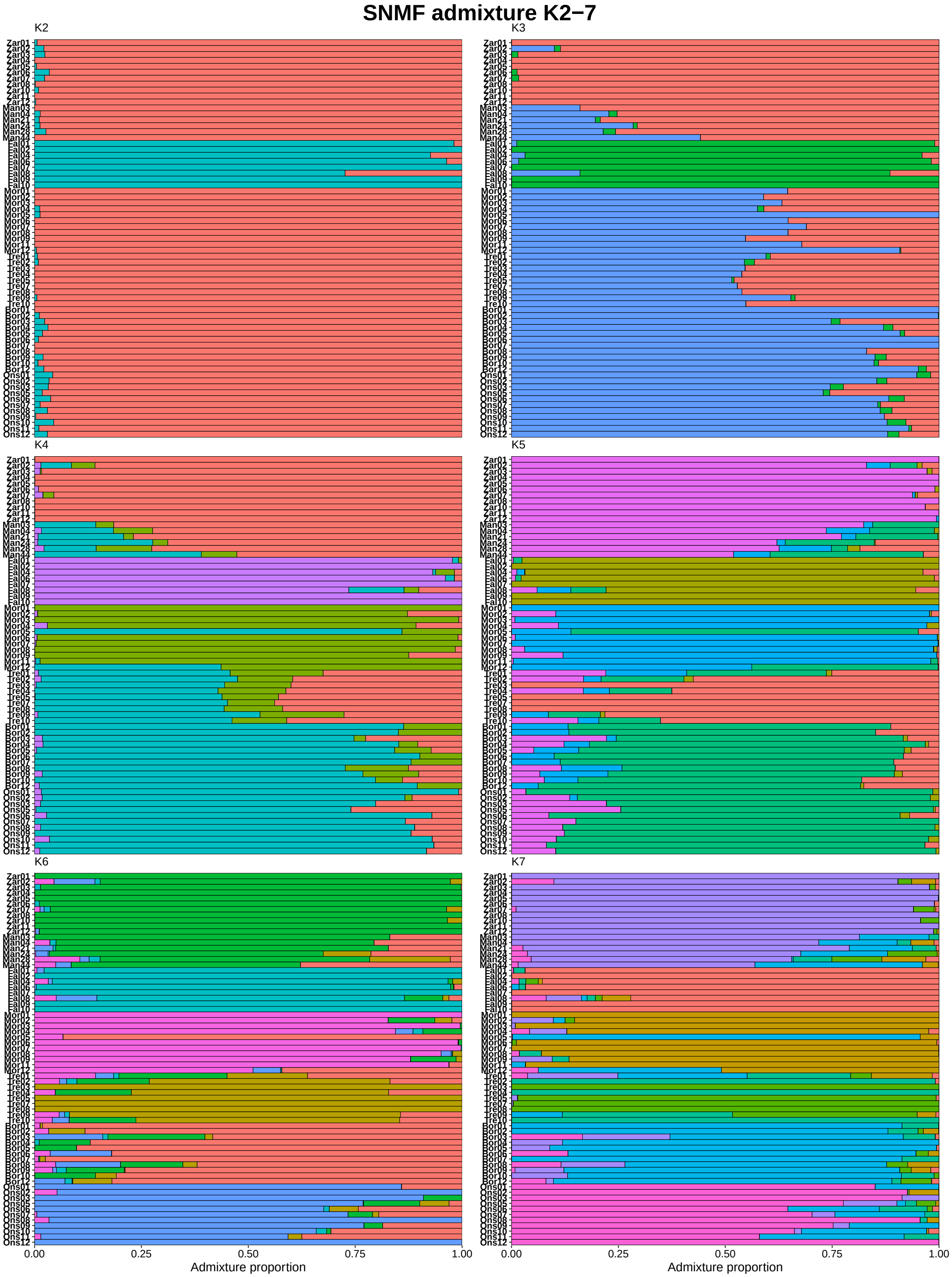

Figure S4. (a) Cross-entropy analysis. (b) Sparse non-negative matrix factorisation (SNMF) analysis. Admixture results are shown for K2-7.

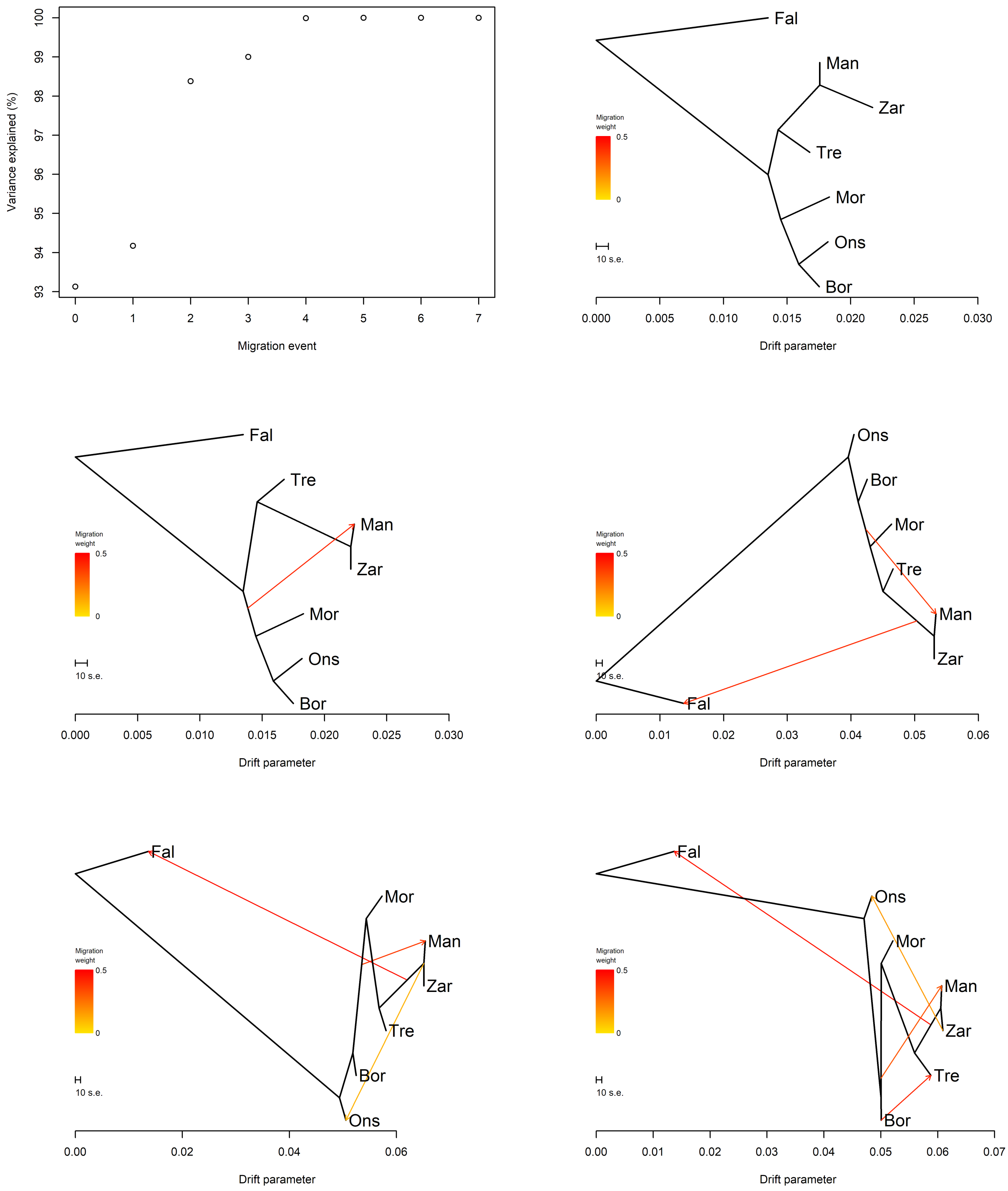

Figure S5. TreeMix results. The top-left plot shows the variance explained (%) in each tree model as migration events are added. The remaining plots show the topology of each model as each migration event is added.

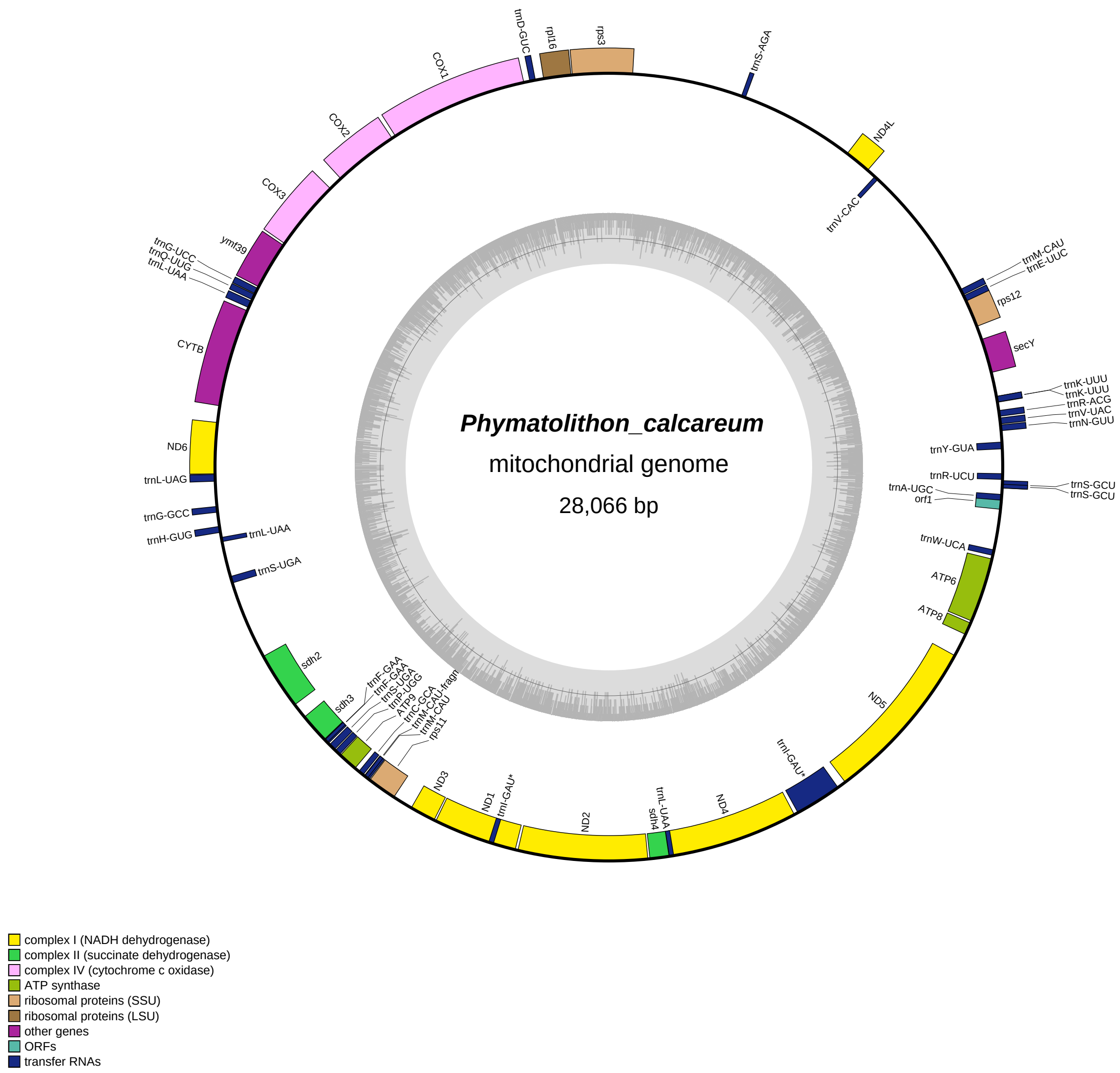

Figure S6a. Tre04 mitogenome annotation. GeSeq was used for gene prediction and annotation using The Mold, Protozoan, and Coelenterate Mitochondrial Code and the Mycoplasma/Spiroplasma Code. ARWEN 1.2.3 was used to annotate tRNAs.

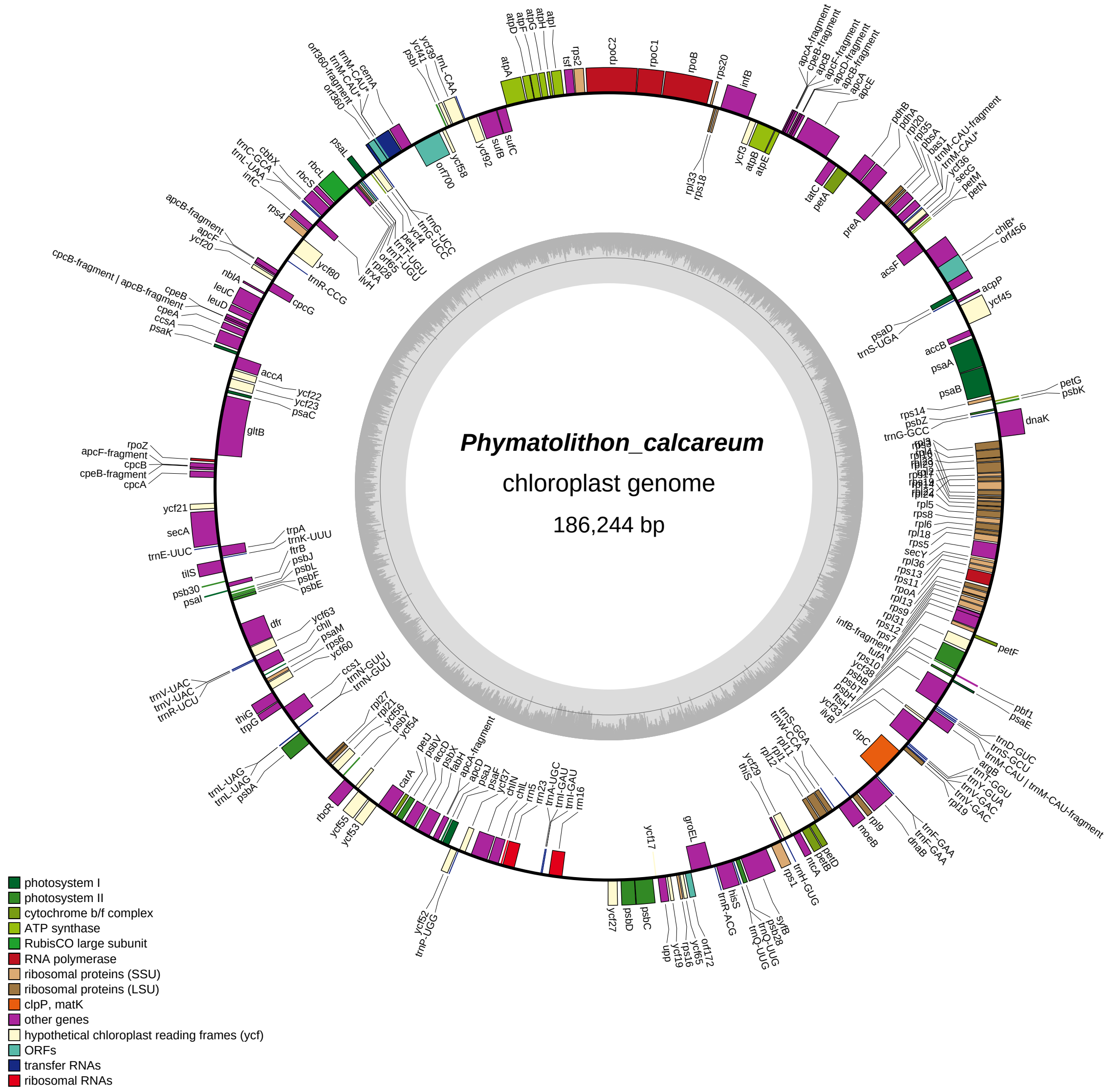

Figure S6b. Tre04 plastome annotation. GeSeq was used for gene prediction and annotation using The Bacterial, Archaeal and Plant Plastid Code. ARWEN 1.2.3 was used to annotate tRNAs.

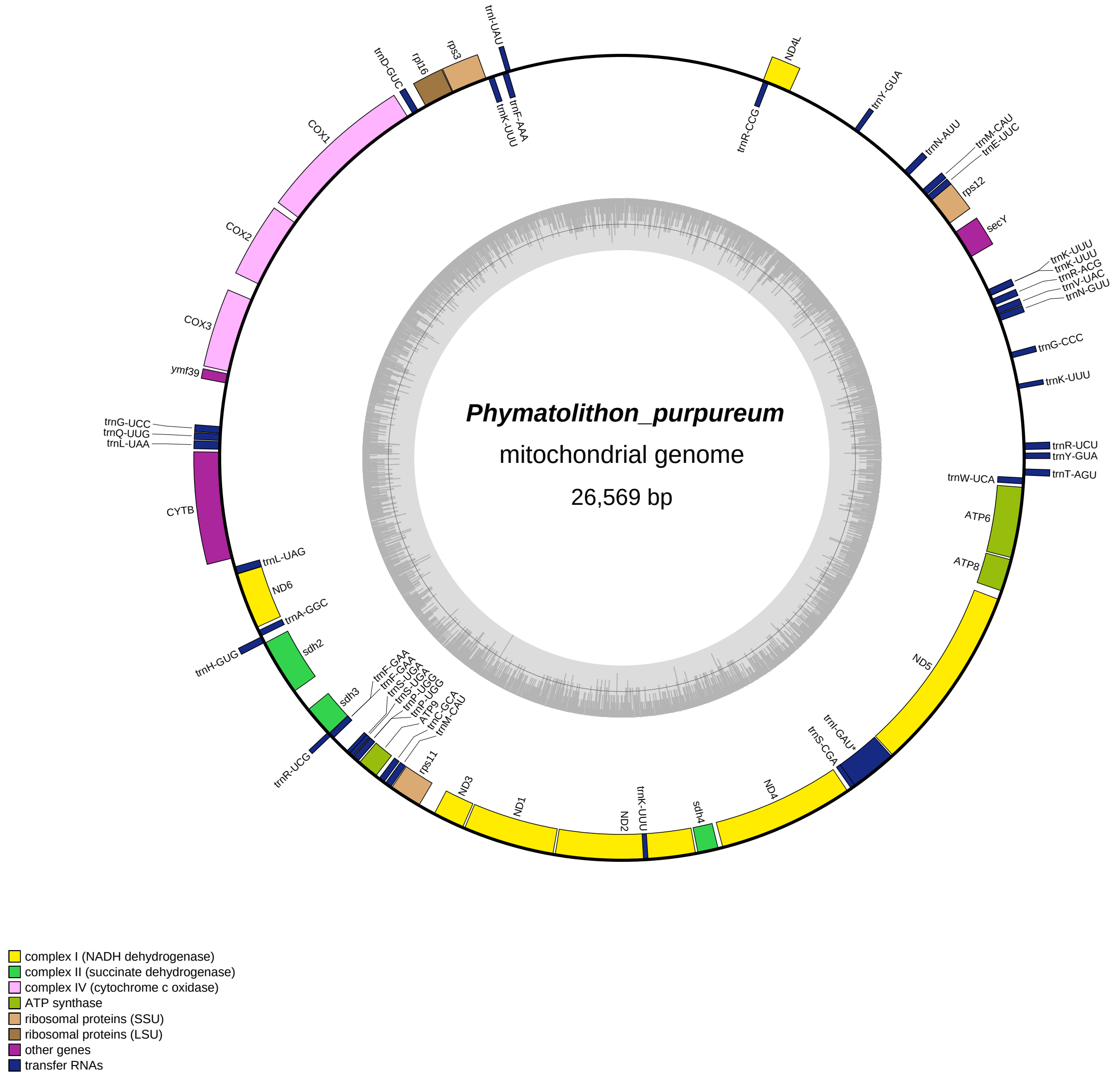

Figure S6c. Sco01 mitogenome annotation. GeSeq was used for gene prediction and annotation using The Mold, Protozoan, and Coelenterate Mitochondrial Code and the Mycoplasma/Spiroplasma Code. ARWEN 1.2.3 was used to annotate tRNAs.

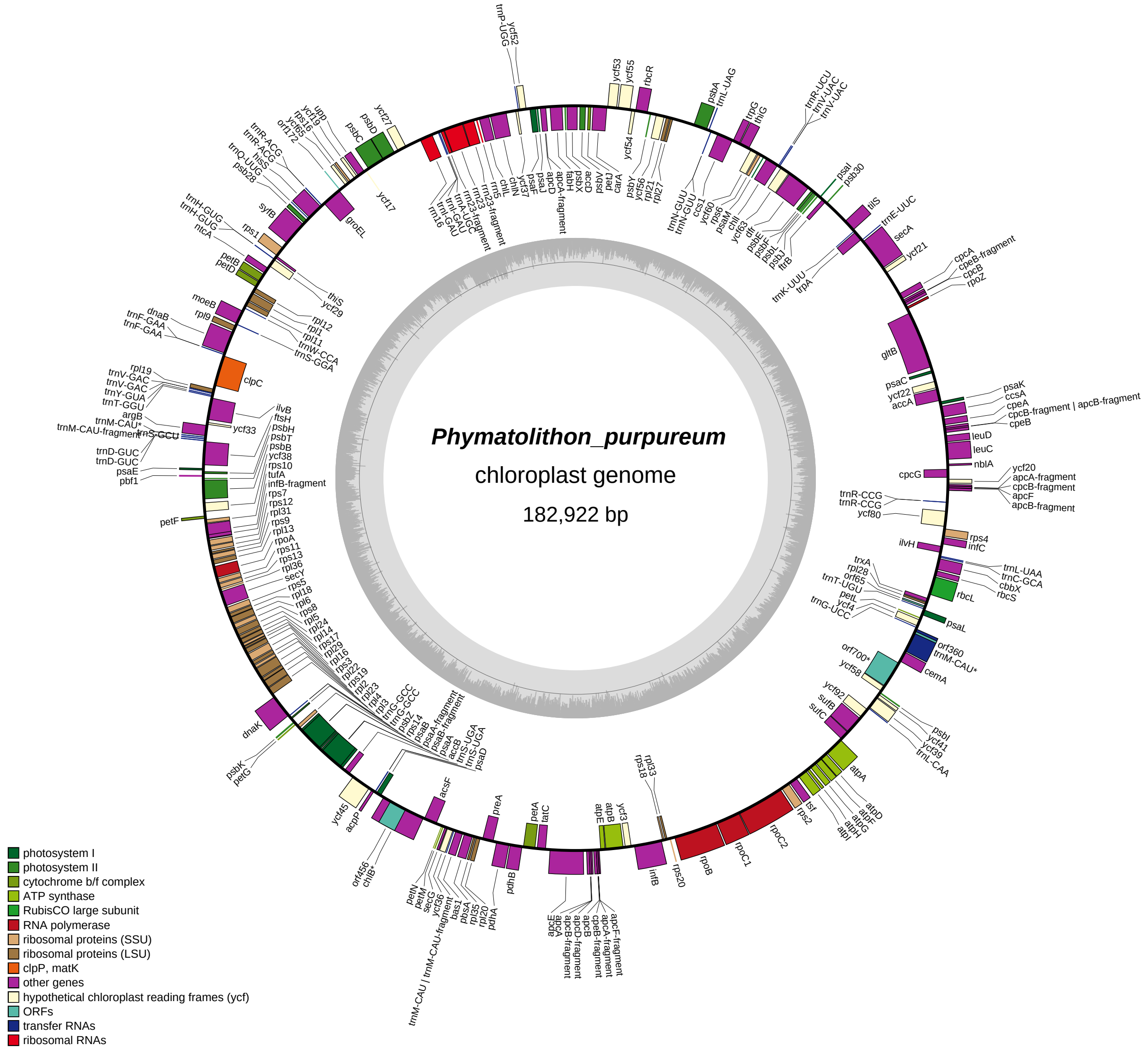

Figure S6d. Sco01 plastome annotation. GeSeq was used for gene prediction and annotation using The Bacterial, Archaeal and Plant Plastid Code. ARWEN 1.2.3 was used to annotate tRNAs.

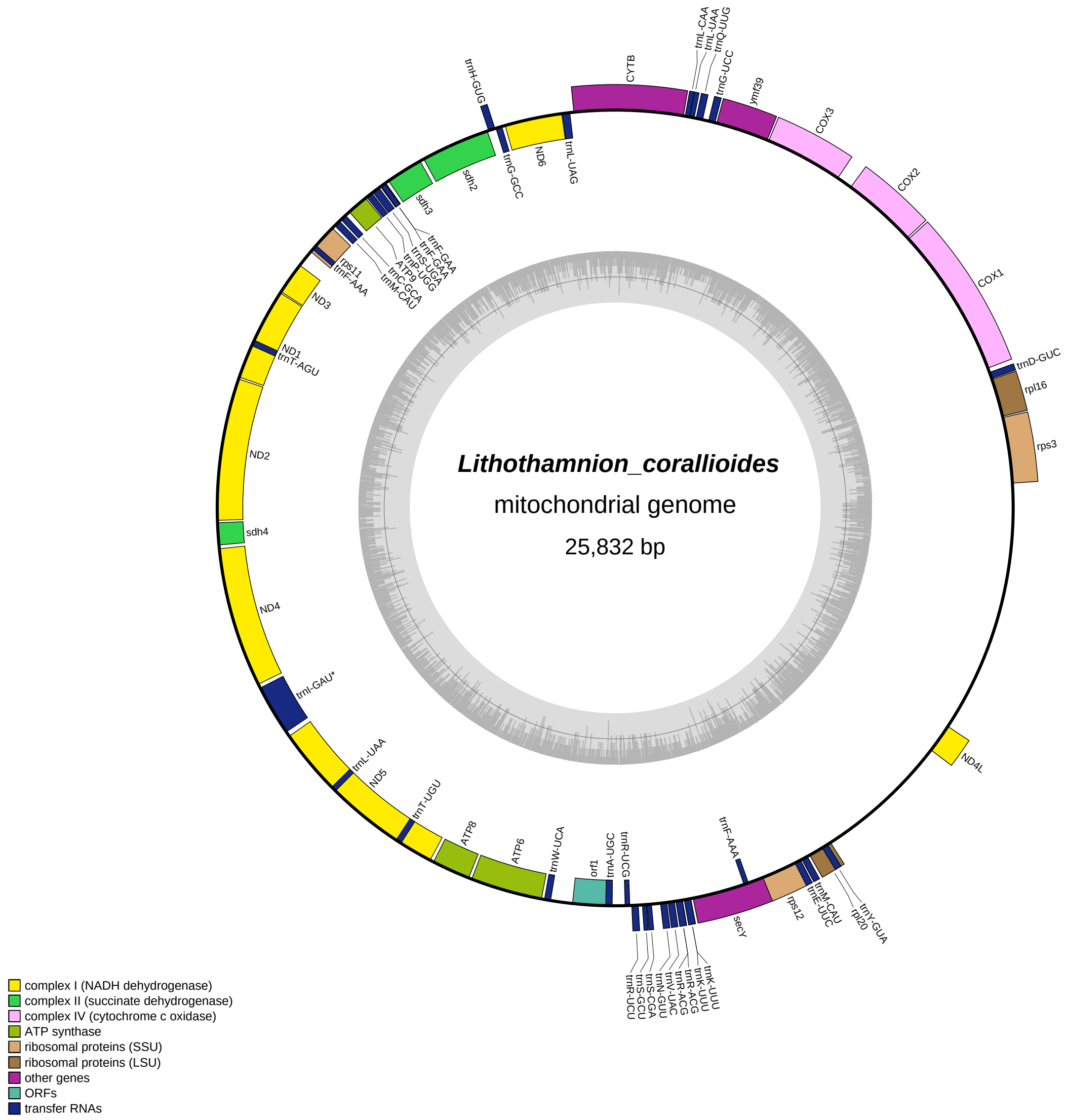

Figure S6e. Lcor09 mitogenome annotation. GeSeq was used for gene prediction and annotation using The Mold, Protozoan, and Coelenterate Mitochondrial Code and the Mycoplasma/Spiroplasma Code. ARWEN 1.2.3 was used to annotate tRNAs.

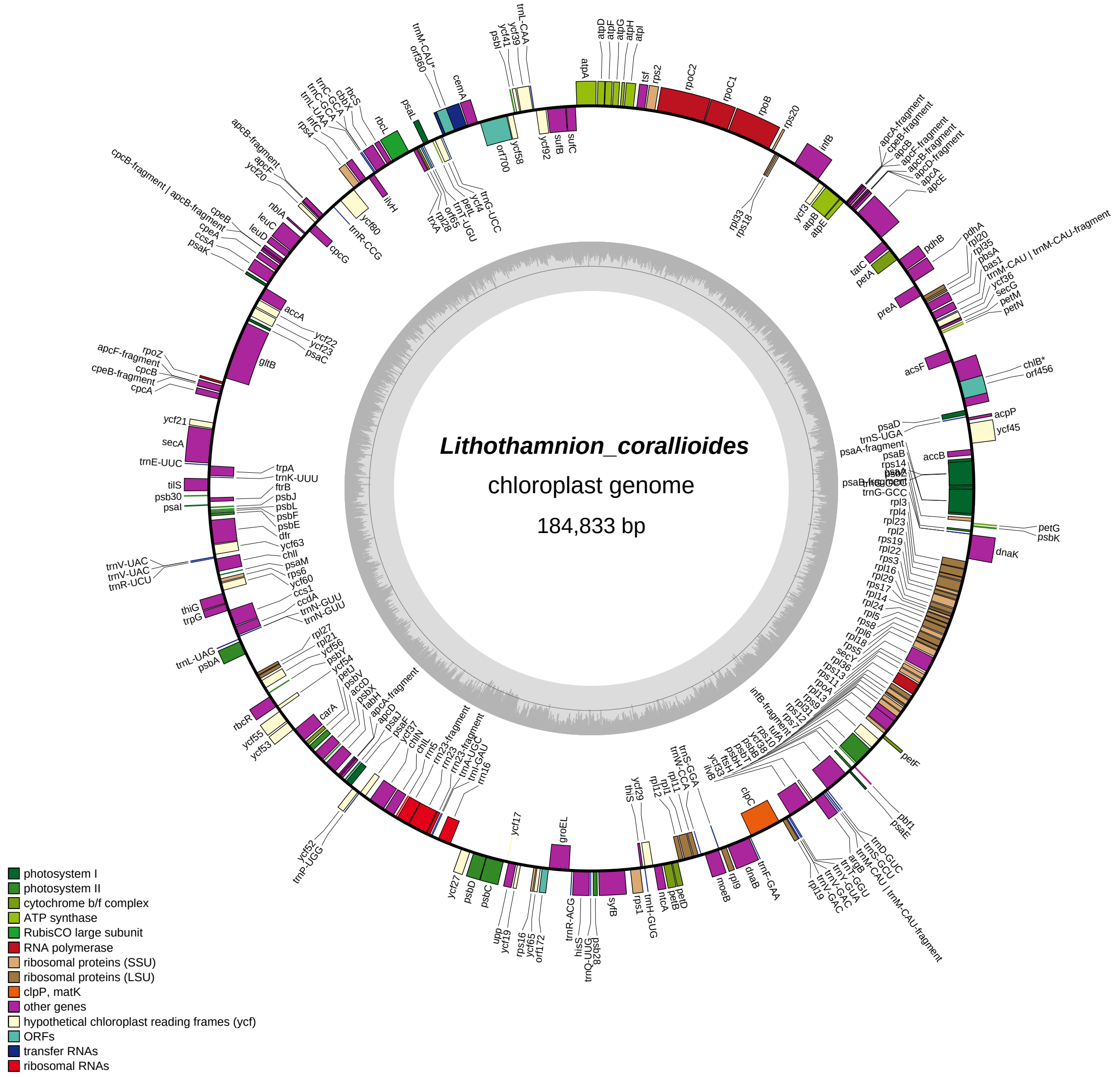

Figure S6f. Lcor09 plastome annotation. GeSeq was used for gene prediction and annotation using The Bacterial, Archaeal and Plant Plastid Code. ARWEN 1.2.3 was used to annotate tRNAs.

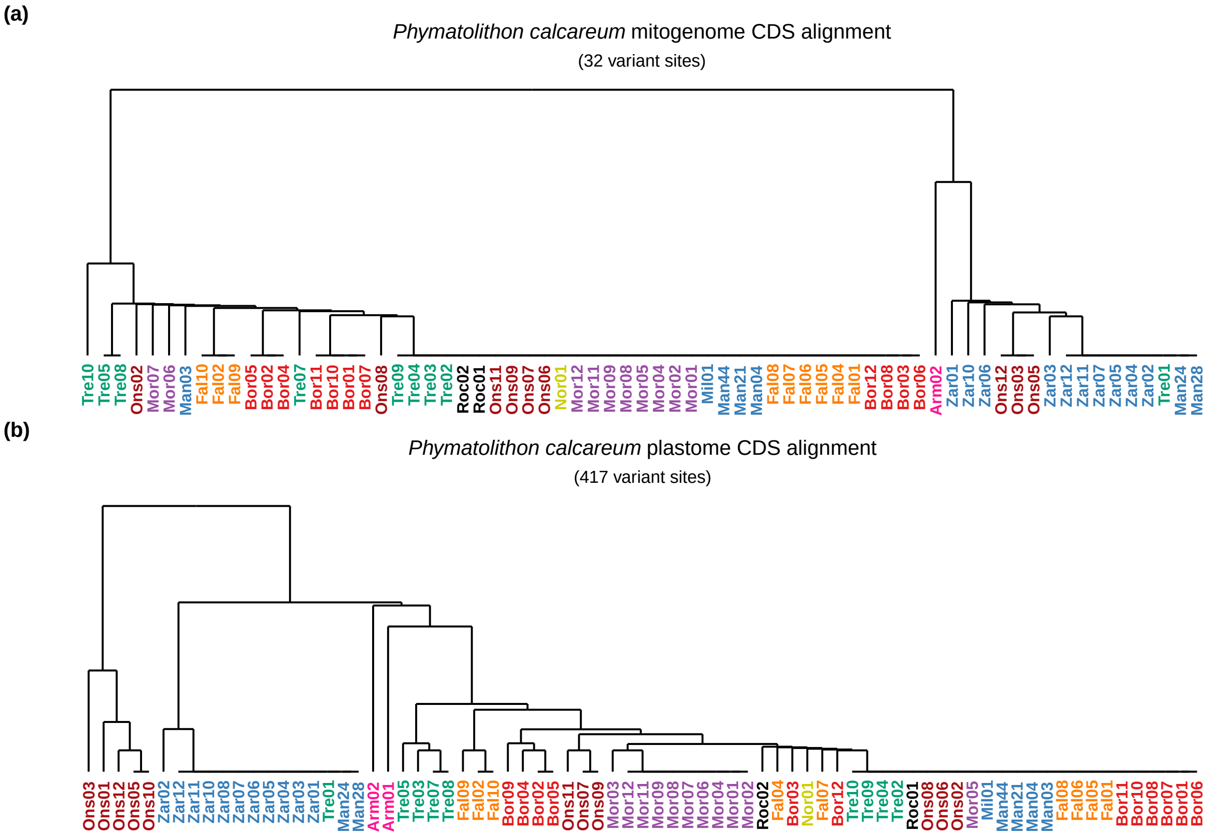

Figure S7. Hierarchical clustering analysis using Tamura and Nei (1993) pairwise genetic distances. (a) Analysis conducted using an alignment of 24 concatenated mitochondrial coding sequences from 69 *Phymatolithon calcareum* individuals. (b) Analysis conducted using an alignment of 212 concatenated chloroplast coding sequences from 75 *P. calcareum* individuals. See Table 1 for code translation and sampling site information.

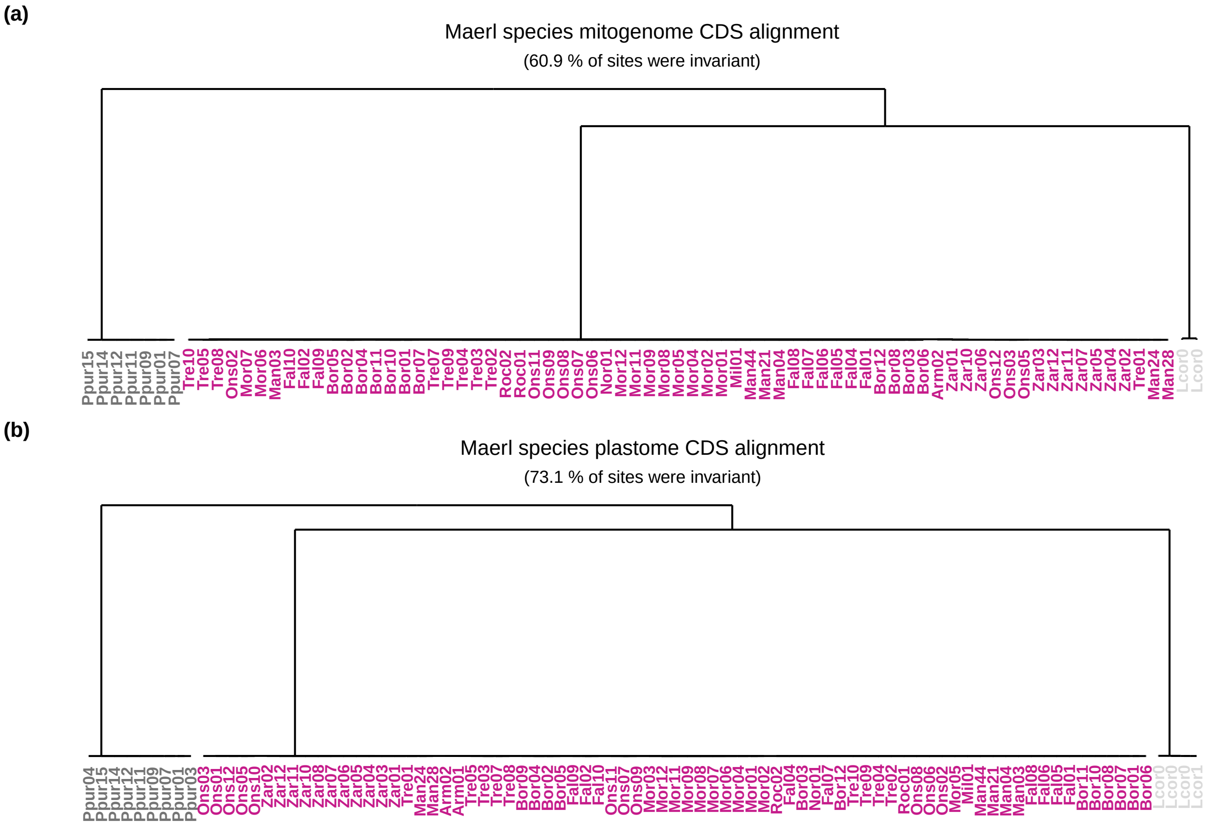

Figure S8. Hierarchical clustering analysis using Tamura and Nei (1993) pairwise genetic distances. (a) Analysis conducted using an alignment of 23 concatenated mitochondrial coding sequences from 69 *Phymatolithon calcareum* individuals, seven *P. purpureum* individuals, and two *Lithothamnion corallioides* individuals. (b) Analysis conducted using an alignment of 212 concatenated chloroplast coding sequences from 75 *Phymatolithon calcareum* individuals, nine *P. purpureum* individuals, and four *Lithothamnion corallioides* individuals. See Table 1 for code translation and sampling site information.
